# A human Staufen1 BAC transgenic mouse exhibits abnormal autophagy and neurodegeneration across the central nervous system

**DOI:** 10.1038/s41419-026-08830-x

**Published:** 2026-05-14

**Authors:** Stefan M. Pulst, Sharan Paul, Hieu Nguyen, Warunee Dansithong, Karla P. Figueroa, Mandi Gandelman, Nancy M. Bonini, Daniel R. Scoles

**Affiliations:** 1 Department of Neurology, University of Utah, 175 North Medical Drive East, 5th Floor, Salt Lake City, UT 84132 USA; 2 Department of Biology, University of Pennsylvania, Philadelphia, PA 19104 USA

**Keywords:** Amyotrophic lateral sclerosis, Alzheimer's disease

## Abstract

RNA-binding proteins (RBPs) play an essential role in development, normal functioning, and human disease. Staufen1 (STAU1) is an RBP that regulates mRNA degradation and subcellular localization, and is part of the ATXN2 protein complex. Previously, we showed that STAU1 is overabundant in patient fibroblasts and in mouse models of Alzheimer’s disease (AD), amyotrophic lateral sclerosis (ALS), and spinocerebellar ataxia type 2 (SCA2), where it is associated with impaired autophagic flux due to STAU1-mediated upregulation of mTOR translation. STAU1 overabundance and impaired autophagy cause accumulation of biomolecular condensates and abnormal unfolded protein response (UPR). We generated a mouse model expressing the entire human *STAU1* gene (h*STAU1*) in a bacterial artificial chromosome (BAC) construct. hSTAU1 in these mice was expressed in cerebral hemispheres, cerebellum, and spinal cord, as well as cultured cortical neurons and cortical and spinal cord astrocytes, and microglia. Expression of hSTAU1 caused dysregulated gene expression, abnormal autophagy, glial activation, and changes in neuronal marker proteins. All of these were significantly improved by reducing STAU1 abundance by RNAi, but exacerbated in BAC-STAU1 mice crossed with Prp-TDP-43(Q331K) transgenic mice. Similar results were also obtained in eye phenotypes in ALS- and SCA2-relevant fly models upon changing staufen-1 dosage. Despite the molecular changes, we observed no overt behavioral changes in mice up to 55 weeks of age, suggesting that STAU1 may function as an epistatic modifier of neuronal degeneration. The BAC-hSTAU1 mouse will be useful for developing therapies targeting the human STAU1 gene.

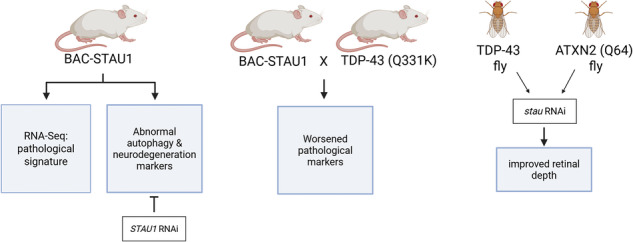

## Introduction

STAU1 is a double-stranded RNA-binding protein (RBP) initially described in the fly for its essential role in the transport and spatiotemporal localization of mRNAs critical for embryonic patterning [1]. It is a multi-functional RBP that also regulates mRNA translation and decay through interactions with double-stranded structures in specific 5′ and 3′ UTRs [2].

STAU1 is an interactor of ATXN2 [3], another RBP that is mutated in spinocerebellar ataxia type 2 (SCA2) [4] and, more rarely, in ALS and PD (reviewed in Scoles and Pulst [5]). STAU1 is also a stress granule (SG) protein that aggregates with ATXN2 in cytoplasmic condensates within cerebellar Purkinje cells (PCs) of SCA2 patients and mice [3]. STAU1 abundance is increased in multiple neurodegenerative disease (NDD) models and in tissues from patients with SCA2, sporadic and familial ALS, and frontotemporal dementia (FTD) associated with C9ORF72 and TDP-43 pathology [3, 6, 7]. In Alzheimer disease and AD related disorders (AD/ADRDs), STAU1 overabundance is seen in APP/PS1 transgenic mice with accumulation of β-amyloid (Aβ) and Tau-neurofibrillary tangles. This was associated with STAU1-mediated stabilization of β-amyloid converting enzyme 1 (BACE1*)* expression [8].

Mechanistically, STAU1 overabundance elevates mTOR translation by directly interacting with the 5’-UTR *MTOR* mRNA. As mTOR is a negative regulator of autophagy, its increased activity leads to autophagy suppression [7]. Thus, STAU1 overabundance is a post-transcriptional effect associated with decreased autophagic clearance due to impaired autophagy [3, 7]. We have proposed a model that leads to a maladaptive feed-forward loop that can be initiated by cellular stress, STAU1 overabundance, and reduced autophagy or misfolded proteins [9]. Recently, Zhou et al. independently confirmed the control of *MTOR* translation by STAU1 and also demonstrated that STAU1 overabundance drives the production of biomolecular condensates rich in *MTOR* mRNA [10]. Furthermore, STAU1 can act as a modulator of the unfolded protein response (UPR): In fibroblasts from individuals with SCA2 or pathogenic TDP-43 or C9ORF72 mutations, increased CHOP levels were restored to normal by STAU1 knockdown [11].

Other functions of STAU1 include degradation of cell-specific subsets of mRNAS, usually by interaction with the 3’-UTR in a process designated Staufen1-mediated decay (SMD), a UPF1-dependent process linked to cell death [12, 13]. In neurons, STAU1 traffics mRNAs in coordination with TDP-43, implicating it in synaptic function, cognitive decline, and autophagy-related neuroprotection [14–18]. STAU1 also binds Alu and IRAlu elements to control mRNA nuclear export [19–21].

The multi-facetted role of STAU1 in NDDs prompted us to study whether STAU1 was necessary and/or sufficient to mediate changes in autophagy or in UPR in cells with NDD-causing mutations. Indeed, in vitro STAU1 knockdown normalized these cellular processes. Surprisingly, exogenous expression of STAU1 in wildtype cells was sufficient to alter autophagy and UPR [7, 11].

Here we further extended these studies for understanding the consequences of STAU1 overabundance in vivo. To this end, we generated a new bacterial artificial chromosome (BAC)-STAU1 mouse that includes 133.8 kb of the human STAU1 locus including all exons and introns as well as up- and downstream regions. This mouse line will also be useful for preclinical development of human *STAU1*-directed therapies.

## Materials and methods

### Mice

The BAC-STAU1 mouse model was developed by the University of Utah Transgenic Mouse Core by pronuclear microinjection of a non-linearized human Staufen1 bacterial artificial chromosome (BAC) construct (BAC-STAU1 clone RP11-120I11, BACPAC Resources) into fertilized oocytes sourced from mice with a B6/D2 mixed hybrid background (B6D2F1J, The Jackson Laboratory stock #100006). Prior to injection, the BAC construct was separated from other nucleotide fragments by pulsed field gel electrophoresis, and gel purified, by the University of Michigan Transgenic Mouse Core. Genotyping of mouse tail DNA was initially done with multiplex PCRs covering all coding exons, 5’ and 3’ UTR, and the 5’ upstream promoter region (ENST00000371856.7, STAU1-207, NM_017453.4). Transgenic mouse bone marrow samples were sequenced and analyzed by Cergentis to determine integration sites and vector-vector breakpoints that represent concatemerization. BAC-STAU1 mice were maintained in a B6D2 mixed background by backcrossing to B6D2F1J no less than every 4 generations. The *Stau1*^*tm1Apa(-/-)*^ (*Stau1*^*-/-*^) mouse [22] was a generous gift from Prof. Michael A. Kiebler, Ludwig Maximilian University of Munich, Germany, and maintained in a C57BL/6 J background. Mice were maintained in a temperature and humidity-controlled environment on a 12 h light/dark cycle with light onset at 6:00 AM. Both males and females were used in animal studies.

### Genotyping

Genotyping was performed by PCR using tail DNAs as previously described [23]. Our approach verified the presence of all *STAU1* exons. Genotyping primers are provided in Supplemental Table 1.

### Immunofluorescent labeling

To perform histological analysis, 4 WT and 4 BAC-STAU1 mice were perfused with PBS, after which brains were dissected and post-fixed in 4% paraformaldehyde (PFA) for 24 h. Tissue was paraffin-embedded and sectioned at the ARUP Laboratories Research Histology Core. Four sections from each mouse were deparaffinized and rehydrated using xylene, ethanol, water, and phosphate buffered saline (PBS), followed by antigen retrieval in boiling citric acid–based antigen unmasking solution for 10 min (Vector Laboratories, H-3300-250). Tissues were permeabilized with 0.3% Triton X-100 for 15 min and blocked in 2% goat serum with 0.01% Triton X-100 for 2 h. Sections were incubated overnight with anti-STAU1 primary antibody (Boster, PB9895; 1:400 dilution in blocking solution), followed by incubation with goat anti-rabbit Alexa Fluor™ Plus 594 secondary antibody (Thermo Fisher Scientific, A32740; 1:1500 dilution). Slides were mounted using ProLong Gold antifade mountant (Thermo Fisher Scientific, P36934). Imaging was performed using a Zeiss AxioScan 7 at the Cell Imaging Core, University of Utah. Quantification of fluorescence was performed using QuPath v0.6.0 [24], with regions manually selected for analysis and cell detection and intensity measurements applied uniformly across all images with the automated scripts feature. Mean fluorescence values for each genotype were compared using a *t* test.

### Primary cell culture

Purified astrocytes and microglia were prepared from mixed glial cultures from spinal cord or brain cortex of neonatal mice as previously described [25, 26]. Mice were sacrificed and brain cortices and spinal cords were isolated for culture and cerebella for genotyping by PCR as described above. To establish primary mixed glial cultures, the corresponding tissue was dissected, digested with trypsin, mechanically dissociated and passed through a 50 µM mesh. The single cell suspension was plated at a density of 2 × 10^4^ cells/cm^2^ and maintained in DMEM supplemented with 10% fetal bovine serum, penicillin and streptomycin (reagents from ThermoFisher). When the astrocyte monolayers were confluent, the cultures were vigorously tapped to dislodge microglia growing on top. The microglia were collected, seeded into a new culture dish and maintained in the same culture media. Astrocytes were further purified by adding 5 µM cytosine arabinoside (Thermofisher) for 24 h. Samples were collected after 2 weeks in culture by lysing cells directly in Laemmli buffer with 5% β-mercaptoethanol (Bio-Rad) for western blot, or in RLT buffer (Qiagen) for qPCR.

Primary cortical neuron cultures were prepared from neonatal mouse cortices, and cerebella were used for genotyping by PCR. Cortices were isolated, digested with a papain enzyme kit (Worthington) according to the manufacturer’s instructions, mechanically dissociated and passed through a 50 µM mesh. The single cell suspension was plated at 5 × 104 cells/cm^2^ in a substrate of poly-l-ornithine and laminin in Neurobasal Plus media supplemented with 2% B27 Plus and 500 mM glutamax (reagents from ThermoFisher). After 72 h cultures were incubated with 1 µM cytosine arabinoside for 24 h to eliminate glial cells. Samples for western blots were collected after 2 weeks in culture by lysing cells directly in Laemmli buffer with 5% β-mercaptoethanol.

### Quantitative PCR (qPCR)

qPCR assays with mice included cDNA preparations using the Life Sciences cDNA Kit (#4368814). Taqman qPCR kits included ThermoFisher Hs00244999_m1 for human *STAU1*, Thermofisher Hs01060665_g1 for human *ACTB*, ThermoFisher Mm00488465_m1 for mouse *Stau1* and Thermofisher Mm01205647_g1 for mouse *Actb*. Taqman assays were performed using Taqman master mix (ThermoFisher 4440040).

### Behavioral phenotype testing

Forelimb and hindlimb grip strength was determined using a grip strength meter (Ugo Basile cat# 47200). Peak force grip strength values are reported as the mean of three replicates. Rotarod testing was performed using the Rotamex-5 accelerating rotarod (Columbus Instruments). In any week of testing, on day 1 mice are handled in the palm of the hand for at least 5 min each for 3 separate times to acclimate animals to the investigator. On day 2, mice are trained on the rotarod with acceleration of 1 RPM per 15 s to a maximum of 10 RPM and total time of 10 min, and this is repeated three times. On days 3–5 mice are placed on the rotarod set to accelerate 1 RPM per 9 s until mice fall from the rod and the latency to fall (in seconds) is recorded, and 2 trials per day are performed. Each mouse group included 20 or more animals, exceeding the minimum sample size required to achieve 80% power to detect a 50% difference between groups, based on power analysis performed using nQuery Advisor. The analysis assumed a two-sided test with α = 0.05. No mice were subjected to exclusion. The technician was blinded to the genotype when performing motor phenotype tests.

### Western blot analyses

Protein extracts from mouse cerebellar or cerebral hemisphere or spinal cord tissues were prepared by homogenization in an extraction buffer (25 mM Tris-HCl pH 7.6, 300 mM NaCl, 0.5% Nonidet P-40, 2 mM EDTA, 2 mM MgCl_2_, 0.5 M urea. and protease inhibitors; Sigma; cat# P-8340). The homogenates were subjected to sonication (3 s for 1 stroke, and level 2; Sonic Dismembrator, Fisher Scientific) followed by centrifugation at 4 °C for 20 min at 14,000 rpm. Only the supernatants were used for Western blotting. Protein extracts were resolved by SDS-PAGE and transferred to Hybond P membranes (Amersham Bioscience Inc., USA). After blocking with 3% skim milk in 0.1% Tween 20/PBS, the membranes were incubated with primary antibodies in 3% skim milk in 0.1% Tween 20/PBS for 2 h at room temperature or overnight at 4 °C. After washing in 0.1% Tween 20/PBS, the membranes were incubated with the corresponding secondary antibodies conjugated with HRP in 3% skim milk in 0.1% Tween 20/PBS for 2 h at room temperature and washed again. Signals were detected by using the Immobilon Western Chemiluminescent HRP Substrate (Millipore Inc., USA; cat# WBKLSO100) according to the manufacturer’s protocol, and detected using a ChemiDoc System (Bio-Rad). Band intensities were quantified by ImageJ software analyses after inversion of the images and proteins were quantitated as a ratio to β-actin (ACTB) or glyceraldehyde-3-phosphate dehydrogenase (GAPDH). We used Precision Plus Protein Dual Color Standards (Bio-Rad Inc., USA, Cat# 1610374) for SDS-PAGE analyses. All proteins were normalized to ACTB except cleaved CASP3 (cCASP3) that was normalized to GAPDH.

### Antibodies

The STAU1 antibody used for western blotting was rabbit polyclonal anti-Staufen (Novus Biologicals, NBP1-33202). We previously validated antibody specificity by overexpression and siRNA-mediated knockdown of STAU1 in cultured cells, followed by immunoblot analysis demonstrating concordant changes in band intensity at the expected molecular weight [3, 11]. The STAU1 antibody used for immunofluoescent labeling was rabbit polyclonal anti-STAU1 (Boster Biological Technology, PB9895). Other antibodies in the study included rabbit polyclonal anti-mTOR antibody (Cell Signaling Technology, 2972); rabbit polyclonal anti-Phospho-mTOR (Ser2448) [(1:3000), Cell Signaling Technology, 2971]; rabbit polyclonal anti-SQSTM1/p62 antibody (Cell Signaling Technology, 5114); rabbit polyclonal anti-LC3B antibody (Novus Biologicals, NB100-2220); mouse monoclonal anti-TDP-43 (human specific) antibody [(1:7000), Proteintech Group, Inc., (Cat #60019-2-Ig)]; rabbit polyclonal human/mouse anti-TDP-43 antibody [(1:7000), Proteintech Group, Inc., (Cat #10782-2-AP)]; mouse monoclonal anti-Calbindin-D-28K antibody [(1:5000), Sigma-Aldrich, C9848]; rabbit polyclonal anti-RGS8 antibody [(1:5000), Novus Biologicals, NBP2-20153]; mouse monoclonal anti-PCP2 antibody (F-3) [(1:3000), Santa Cruz, sc-137064]; rabbit polyclonal anti-PCP4 antibody [(1:5000), Abcam, ab197377]; Phospho-p70 S6 Kinase (Thr389) antibody [(1:3000), Cell Signaling, Cat# 9205]; GFAP (GA5) mouse mAb [(1:7000), Cell signaling, Cat #3670]; ChAT (E4F9G) Rabbit mAb [(1:5000), Cell Signaling, Cat# 27269]; NeuN (D4G4O) XP® Rabbit mAb [(1:5000), Cell Signaling, Cat# 24307]; GAPDH (14C10) rabbit mAb [(1:7,000), Cell Signaling, Cat# 2118]; Cleaved Caspase-3 (Asp175) (5A1E) rabbit mAb [(1:3000), Cell Signaling, Cat #9664]; Anti-NeuN107B antibody [(1:5,000)] Abcam, (ab175148); GFP (D5.1) rabbit mAb [(1:6,000), Cell Signaling, Cat #2956]; UNC13A/Munc13-1 polyclonal antibody [(1:3000), Proteintech Group, Inc., Cat #55053-1-AP]; STMN2 polyclonal antibody [(1:3000), Proteintech Group, Inc., Cat #10586-1-AP], and mouse monoclonal anti-β-Actin−peroxidase antibody (clone AC-15) [(1:10,000)], (Sigma-Aldrich, A3854). Secondary antibodies included anti-mouse IgG, HRP-linked antibody [(1:5000), Cell signaling, Cat #7076], and peroxidase AffiniPure goat anti-rabbit IgG (Jackson ImmunoResearch Laboratories, 111-035-144). Secondary antibody used for immunofluorescent labeling was goat anti-rabbit Alexa Fluor™ Plus 594 (Thermo Fisher Scientific, A32740).

### Validation of anti-STAU1 antibody

HEK-293 cells were transfected with siControl (Qiagen, 1027280) or human siSTAU1 (5’-CCUAUAACUACAACAUGAGdTdT-3’), as previously described [3]. At 4 days post-transfection protein extracts were evaluated by western blotting using a 1:5000 dilution of anti-STAU1 antibody (Boster Biological Technology, PB9895).

### RNA-seq

Total RNA was extracted from tissues using the RNeasy Mini-Kit (Qiagen) according to the manufacturer’s protocol. RNA quality was determined using the Agilent ScreenTape Assay. Library preparation was performed using the Illumina TruSeq Stranded Total RNA library prep Ribo-Zero gold. Paired-end 150 bp reads were generated on a Novaseq 6000 S2 cell sequencing instrument at the High-Throughput Genomics and Bioinformatic Analysis Shared Resource at Huntsman Cancer Institute (University of Utah). The human GRCh38 genome and gene annotation files were downloaded from Ensembl release 100 and a reference database was created using STAR version 2.7.3a with splice junctions optimized for 150 base pair reads [27]. Optical duplicates were removed from the paired end FASTQ files using clumpify v38.34 [28] and reads were trimmed of adapters using cutadapt 1.16 [29]. The trimmed reads were aligned to the reference database using STAR in two pass mode to output a BAM file sorted by coordinates. Mapped reads were assigned to annotated genes using featureCounts version 1.6.3 [30]. The output files from cutadapt, FastQC, FastQ Screen, Picard CollectRnaSeqMetrics, STAR and featureCounts were summarized using MultiQC to check for any sample outliers [31]. Differentially expressed genes were identified for siSTAU1 vs siControl using a 5% false discovery rate with DESeq2 version 1.26.0 [32]. Pathways were analyzed using the fast gene set enrichment package with a 10% false discovery rate (FDR) [33, 34]. Replicate RNA-seq experiments were performed independent of one another four years apart with RNAs prepared by different technicians.

### AAV production and treatment of mice

Artificial microRNAs targeting *STAU1* were designed following the method described by Boudreau and Davidson, which begins with siRNA sequences [35]. Two different miRNAs were designed that target human *STAU1* that were based on siRNA sequences that were previously determined effective for lowering STAU1 abundance. One of these siRNAs, developed by us (si*STAU1*, 5’-GUAACUGCCAUGAUAGCCCGAG-3’), was used to design mi*STAU1*-A, and a second was based on a published *STAU1* siRNA (5’-CCUAUAACUACAACAUGAG-3’) used to design mi*STAU1*-B [36]. PHP.eB serotype AAVs were generated by the Virus Packaging Laboratory of the Drug Discovery Core at University of Utah using pUCmini-iCAP-PHP.eB (Addgene #103005). The viral expression vector that we used (pAAV-U6-sgRNA-CMV-GFP, Addgene #85451) includes the U6 promoter to drive miRNA expression and independently expresses GFP under the control of the CMV promoter. BAC-STAU1 mice and wildtype littermates, 20 wks of age, were treated with AAV (9.6E10) viral genomes (VG) or PBS vehicle as indicated, by single intracerebroventricular (ICV) injections to the right lateral ventricle as previously described [37]. Mice were sacrificed at 23 wks of age when spinal cords were collected for analysis by western blotting.

### *Drosophila* studies

Fly stocks were maintained on standard cornmeal molasses agar. Transgenic lines included gmr-GAL4 (BDSC_1104); UAS-*stau*.RNAi (shRNA) (BDSC_82965); UAS-hTDP-43 (BDSC_79587) [38]; UAS-hATXN2(CAG)64 [39]; UAS-*stau* (*Drosophila* *stau*) [40]. External eye microscopy, paraffin sectioning, and quantification were performed as described [39, 41]. Stau knockdown in these lines has been verified [42]. The GMR-Gal4 functions as a driver line that expresses the Gal4 transcriptional activator under control of the Glass Multimer Reporter (GMR) element. GMR is active in the developing and mature fly eye allowing targeted eye gene expression.

### Statistical analysis

Statistical differences between selected groups evaluated by qPCR, western blotting, and the electrophysiological data were evaluated using blocked two-way analysis of variance (ANOVA) tests followed by post-hoc tests of significance (Tukey’s or Bonferroni’s tests for multiple comparison, as indicated). Data shown on western blots are mean and SD of biological replicates (*n* = 3–4 mice) that were each evaluated on a minimum of three blots (3 technical replicates). Behavioral tests were evaluated with repeated measures ANOVA in GraphPad Prism (grip strength testing) or STATA (rotarod testing). Student’s *t* tests were unpaired and two-tailed. All tests were performed using the GraphPad Prism software package.

## Results

### Generation of BAC-STAU1 mice

**Fig. 1:**
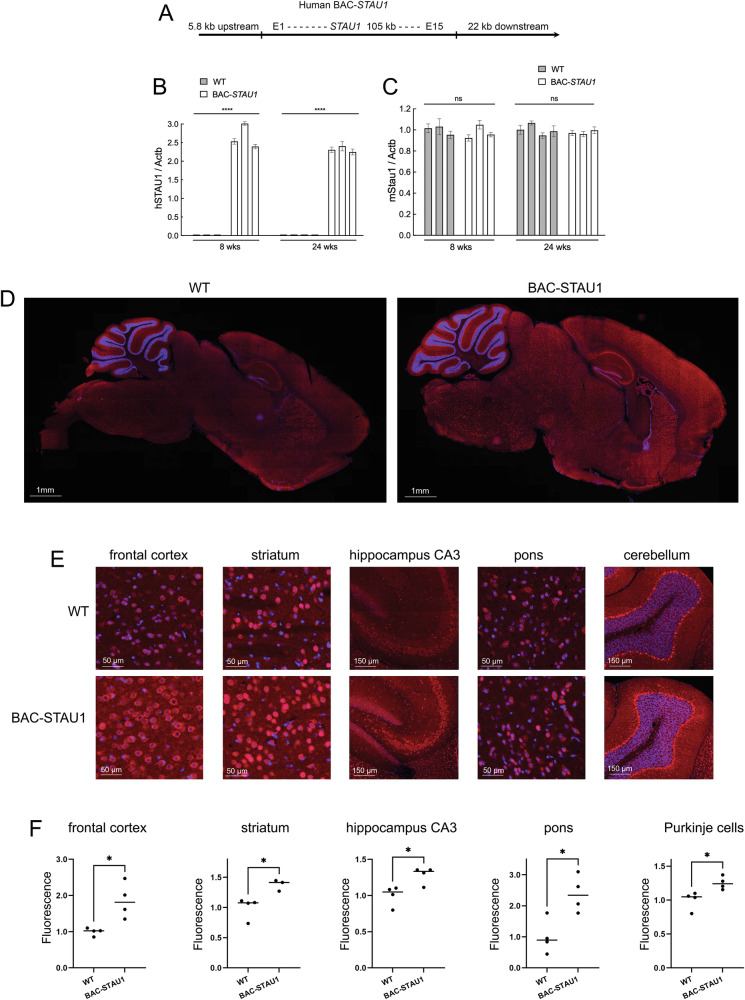
Generation and characterization of a BAC-STAU1 transgenic mouse model. **A** Schematic of the BAC construct showing the relative position of the *STAU1* gene and flanking sequences. qPCR using brain extracts for human *STAU1* (**B**) and mouse *Stau1* (**C**) for 8 and 24 wk old BAC-STAU1 mice compared to wildtype mice. ns, non-significant; ****, *p* < 0.0001, two-way ANOVA with Bonferroni post-hoc correction. **D**–**F** Immunofluorescence microscopy for STAU1 in BAC-STAU1 and WT mouse brain. **D** Representative images of saggital brain sections and **E** higher magnification images of the regions quantified. **F** Quantification of fluorescence intensity for the indicated brain regions. The mean from 4 mice of each genotype is shown. *, *p* < 0.05, Student’s *t* test.

We produced a BAC mouse harboring the human *STAU1* gene. We utilized a 133.8 kb BAC construct (Fig. 1A) and generated transgenic mice by pronuclear injection. Sequencing of DNA isolated from BAC-STAU1 mouse bone marrow specimens determined that one of four founders, only BAC-STAU1.6, had a single genomic integration of the transgene, on chromosome 17 (chr17:9,394,829- ~ 9,610,924). The integration occurred simultaneously to a 210 kb genomic deletion within this region. Query of the NCBI Reference Sequence Database (RefSeq) confirmed that there are no annotated genes in this region of the mouse genome and therefore no gene disruption is predicted. No structural variants were identified within the integrated construct. Due to the nature of the sequence at the integration site, BAC copy number via this method could not be estimated. BAC-STAU1.6 mice, now simply referred to as BAC-STAU1, are viable, display no abnormal gross morphology or significant segregation distortion. Further sequencing also demonstrated that the *STAU1* isoform expressed in CNS tissues of BAC-STAU1 mice is equivalent to the Ensemble STAU1-203 transcript (ENSMUST00000109236.9), and that it lacks a 6 amino acid insertion in the RNA binding domain 3 (RBD3) known to reduce RNA binding affinity [43].

The mean weights of BAC-STAU1 mice were not significantly different from wildtype littermates up to 80 wks (Supplementary Fig. 1). The presence of the BAC-transgene in brain was demonstrated by quantitative PCR using primers specific for human *STAU1* vs mouse *Stau1* (Fig. 1B, C). (The original qPCR data are presented in Supplementary Table 2).

### Age selection rationale

To assess the emergence and progression of molecular phenotypes in BAC-STAU1 mice, different ages were selected based on assay-specific goals rather than to define a single disease stage. Early time points (3–8 weeks) were used to confirm transgene expression across relevant CNS cell types and to establish baseline molecular changes prior to overt behavioral impairment. Young adult ages (14–25 weeks) were chosen to characterize early biochemical and transcriptomic phenotypes and to assess their persistence in vivo. Later time points (57 weeks) were used to assess transcriptional signatures with aging. Genetic interaction studies were performed at both early and adult ages (8 and 24 weeks) to assess age-dependent effects of STAU1 elevation on TDP-43–associated molecular phenotypes.

### Immunofluorescent labeling

We observed widespread STAU1 expression in BAC-STAU1 mice by immunofluorescent labeling, STAU1 was significantly more abundant in tissues of BAC-STAU1 mice including frontal cortex, striatum, hippocampus CA3, pons, and cerebellar Purkinje cells, compared to WT mice, determined by quantitative immunofluorescent microscopy (Fig. 1D–F). Validation of the anti-STAU1 antibody from Boster is shown on full blots in Supplementary Fig. 2.

### BAC-STAU1 is expressed in multiple CNS cell types in BAC-STAU1 mice

To confirm widespread hSTAU1 expression in the CNS we performed western blotting and qPCR analyses using cultured astrocytes, microglia, and neurons prepared from neonate spinal cord and cortical tissues. hSTAU1 was highly abundant in all cultures from BAC-STAU1 mice vs wildtype littermates (Supplementary Fig. 3A–E). We verified that neuronal cultures expressed NeuN and lacked IBA1, while microglia cultures expressed IBA1 which was absent in neuronal cultures, and we also verified GFAP expression in astrocyte cultures (not shown). There was also approximately fourfold more *hSTAU1* mRNA in cultured astrocytes of the cortex vs spinal cord of BAC-STAU1 mice, determined by qPCR, while mouse *Stau1* mRNA was generally mildly reduced in these cells in BAC-STAU1 vs wildtype littermates (Supplemental Fig. 3F, G).

### Behavioral phenotype testing

We investigated BAC-STAU1 mice for behavioral phenotypes using both accelerating rotarod and grip strength tests. No sustained or progressive behavioral differences in motor strength or coordination were observed between BAC-STAU1 and WT mice. A modest reduction in hindlimb grip strength was detected at 16 weeks of age; however, this difference was not observed at 20 weeks, was not present in forelimb grip strength, and was not accompanied by differences in rotarod performance (Supplemental Figs. 4 and 5).

### Molecular phenotype associated with STAU1 overabundance

#### Transcriptome analysis

**Fig. 2:**
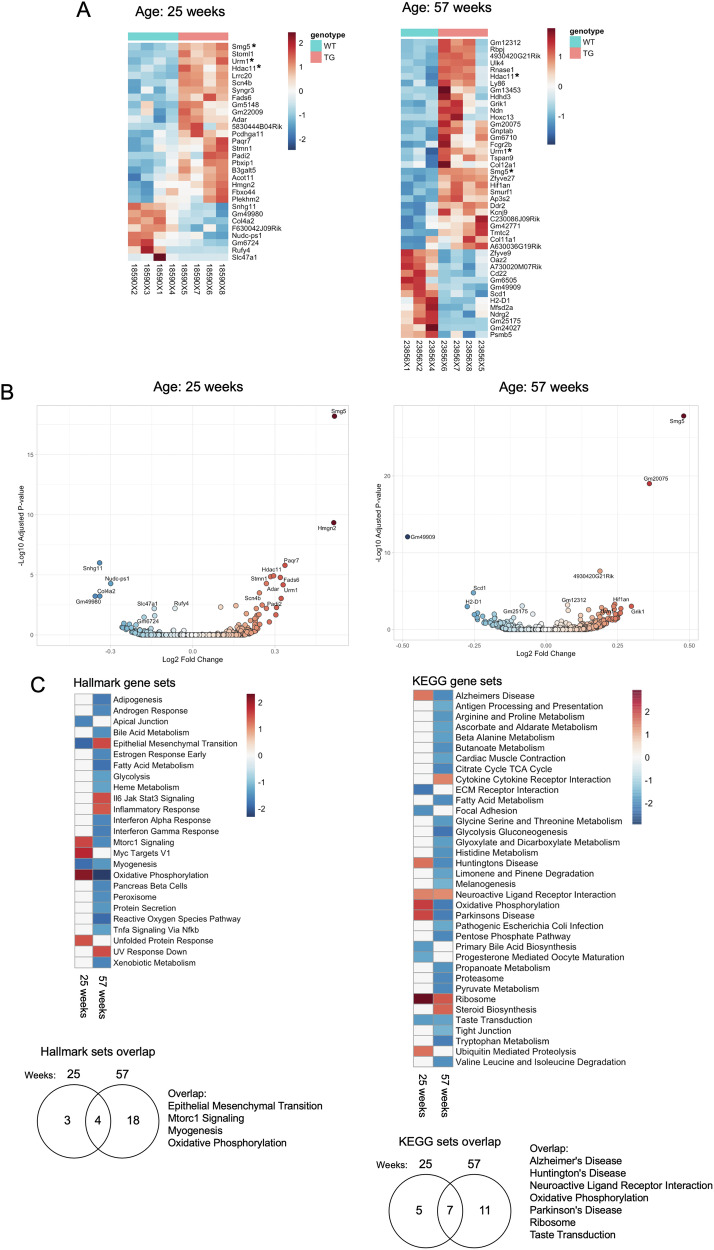
BAC-STAU1 mouse spinal cord transcriptomes. Two independent RNA-seq experiments were performed using 25 wk old and 57 wk old BAC-STAU1 mice. **A** 30 and 44 DEGs were identified (FDR = 10), including 22 up- and 8 downregulated DEGs in the 25 wk group, and 31 up- and 13 downregulated DEGs in the 57 wk group. Three DEGs upregulated in both are marked by an asterisk. **B** Volcano plots showing distribution of up- and downregulated DEGs showing *Smg5* as the most upregulated DEG in both experiments. **C** Hallmark and KEGG pathway analyses showing an increased number of significant annotated pathways in the 57 wk group, and VENN diagrams indicating the numbers of shared pathways between the 25 and 57 wk groups.

We carried out RNA-seq of spinal cords from BAC-STAU1 mice at 25 and 57 weeks of age. Initial analysis at 25 weeks revealed a limited but consistent set of differentially expressed genes, motivating a second analysis that both replicated the experimental design and extended the analysis to an older age to assess age-dependent transcriptomic effects. Rigor of the two experiments is supported by concordant features between the studies, including shared differentially expressed genes and overlapping annotated pathways. The number of mice included were 2 male and 2 female BAC-STAU1 mice compared to 2 male and 2 female WT littermates for the 25 wk group, and 2 male and 2 female BAC-STAU1 mice compared to 2 male and 1 female WT littermates for the 57 wk group. In both experiments the number of differentially expressed genes (DEGs) was low, with an FDR cut-off of 10%. The 25 wk group had 30 DEGs while the 57 wk group had 44 DEGs and 3 were shared including *Smg5*, *Urm1*, and *Hdac11* (Fig. 2A). Volcano plots showed that the most significantly upregulated DEG in both experiments was *Smg5*, a regulator of UPF1 in the nonsense mediated decay (NMD) pathway (Fig. B). Hallmark and KEGG pathway analyses revealed progressive transcriptome dysregulation in BAC-STAU1 mice with age, with an increased number of annotated pathways in older mice. Hallmark analysis showed 7 enriched gene sets in the 25 wk group and 22 in the 57 wk group, while KEGG pathway analysis showed 7 in the 25 wk group and 31 in the 57 wk group (Fig. 2C). Greater numbers of annotated gene sets in the 57 wk group is consistent with a progressive transcriptomic phenotype in BAC-STAU1 mice. Enriched Hallmark gene sets shared between the two groups included Epithelial Mesenchymal Transition, Mtorc1 Signaling, Myogenesis, and Oxidative Phosphorylation. Enriched KEGG gene sets shared between the two groups included Alzheimer’s Disease, Huntington’s Disease, Neuroactive Ligand Receptor Interaction, Oxidative Phosphorylation, Parkinson’s Disease, Ribosome, Taste Transduction. Complete RNA-seq and pathway analyses are provided in Supplemental Table 3.

#### Western immunoblotting for autophagy and pathology markers

**Fig. 3:**
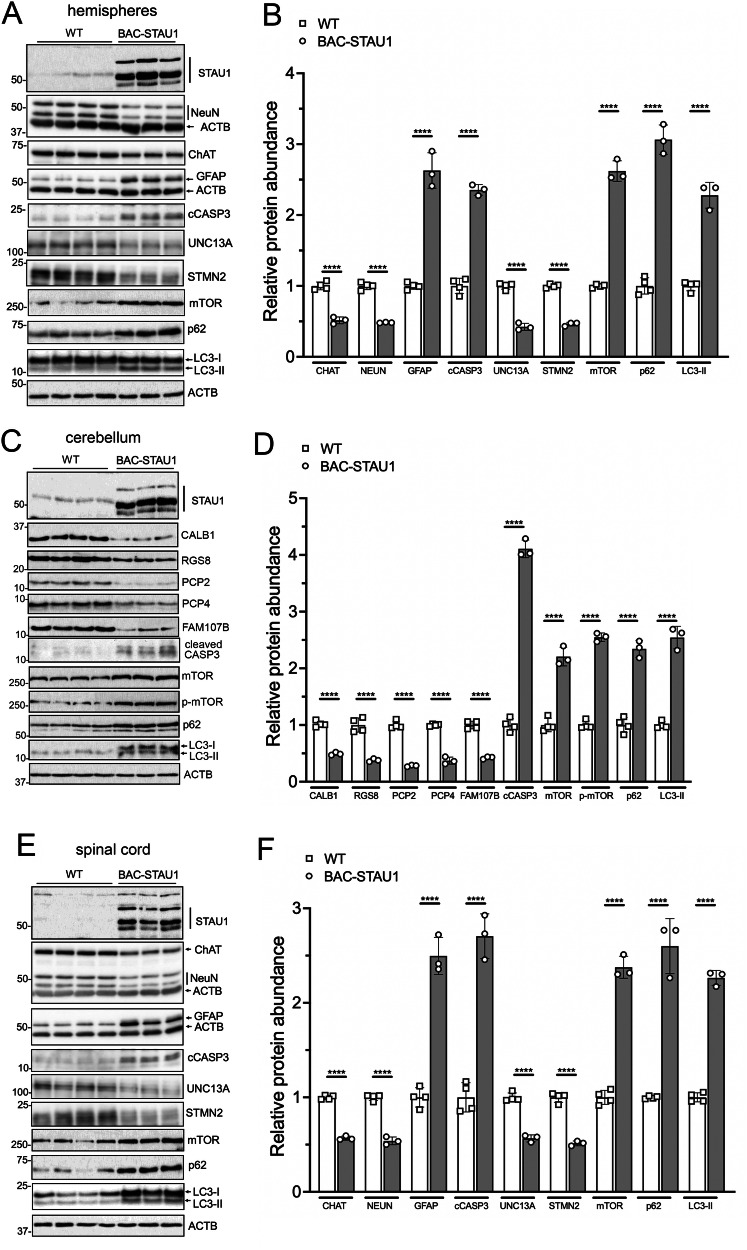
Molecular characterization of BAC-*STAU1* mice. Western blotting showing autophagy and other abnormal molecular marker proteins as indicated in the cerebral hemispheres (**A** & **B**), cerebellum (**C** & **D**) and spinal cord (**E** & **F**). Mice were 14 weeks of age. Each lane represents an individual mouse, *N* = 3–4 mice per group. Means ± SD of biological replicates are plotted; the SD of technical replicates ranged from 0.03–0.54 (**B**), 0.02–0.48 (**D**), 0.05 to 0.37 (**F**). ****, *p* < 0.0001, two-way ANOVA with Bonferroni post-hoc correction.

Given that analysis of DEG pathways showed gene sets in NDDs and MTORC1 signaling, we wanted to test key proteins for their abundance by western blotting. Overabundant STAU1 in the brain, cerebellum, and spinal cord was associated with abnormal abundance of autophagy proteins. These include mTOR, p-mTOR, p62 and LC3-II in the brain, cerebellum, and spinal cord (Fig. 3). The profile of these proteins and their phosphorylated forms are consistent with reduced autophagy, previously described in vitro [3].

We next examined protein changes often associated with neurodegeneration such as reduction of key neuronal marker proteins and an increase in astrogliosis. We isolated protein extracts from cerebral hemispheres, cerebella, and spinal cords of WT and transgenic mice at 14 weeks-of-age and analyzed abundance by semi-quantitative western blot analysis (Fig. 3). In all three tissues, there was a remarkable increase in GFAP and cleaved caspase-3 consistent with increased gliosis and apoptotic cell death. The pan-neuronal marker protein NeuN was decreased in hemispheres and spinal cord, and PC-specific proteins (CALB1, RGS8, PCP2, PCP4, FAM107B) were decreased in cerebellum. Choline-acetyl transferase (ChAT), the marker protein for cholinergic neurons, was decreased by 50% compared to that in WT tissues, not only in spinal cord but also in hemisphere tissue (Fig. 3).

Mis-splicing of specific mRNAs is part of TDP-43 proteinopathy. In human spinal cord, *UNC13* *A* and *STMN2* show cryptic exon splicing in the presence of [44, 45]. Homologous cryptic exons are not known in mice, yet we observed abnormally reduced UNC13A and STMN2 abundance in BAC-STAU1 mouse brain and spinal cord (Fig. 3A, B, E, F).

### Modulation of STAU abundance in flies and mice

We had previously shown that reduction of STAU1 by genetic interaction improved the motor phenotype of SCA2 mice [3]. The results described above suggest that STAU1 overabundance by itself induces a cellular pre-degenerative or risk phenotype. We wanted to test an extension of this hypothesis in fly and mouse models by modulating STAU1 levels in the presence of disease-associated mutations, both reducing and increasing levels compared to wildtype levels.

### Stau dosage modulates retinal degeneration in fly TDP-43 and ATXN2^exp^ models

**Fig. 4:**
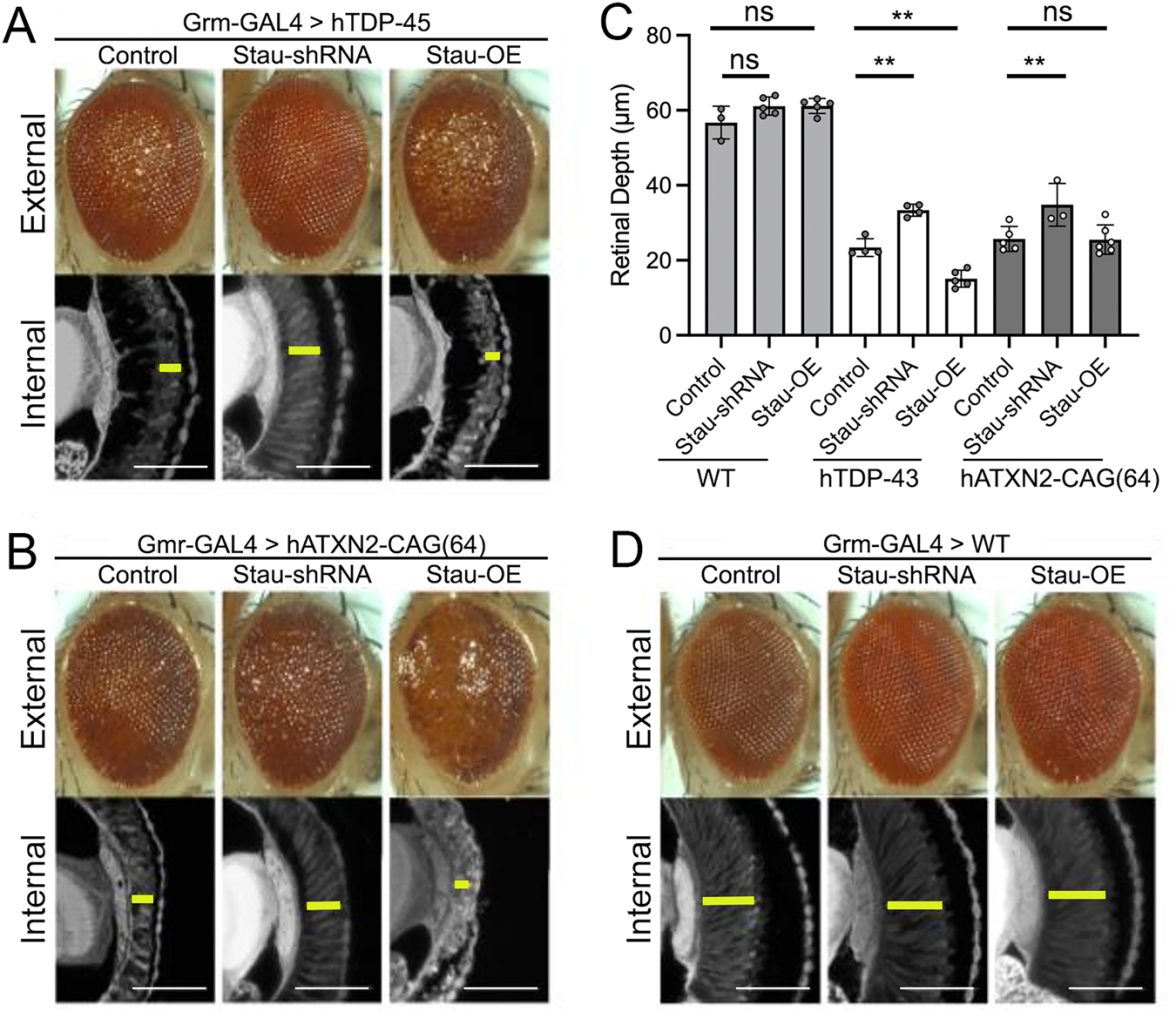
Modulation of fly STAU levels. Upregulation of fly STAU enhances the retinal degeneration phenotype in flies expressing hTDP-43 (**A**) or hATXN2-CAG_64_ (**B**), whereas reduction of Stau by shRNA improves retinal depth. **C** Quantification of retinal depth. **D** Modulation of Stau levels in wildtype (WT) flies has no effect on the external eye or retinal depth eye. Control indicates lines crossed to *w*^*1118*^, a line with normal WT Stau levels. Yellow bars illustrate retinal depth. Scale bars, 85 µm. ns, non-significant; **, *p* < 0.01, two-way ANOVA with Bonferroni post-hoc correction.

We selected fly models expressing human TDP-43 and human ATXN2-CAG_64_ and varied fly *stau* dosage using an upregulation line, and a line that reduced fly *stau* by RNAi. In the presence of normal *stau* levels, both hTDP-43 and hATXN2-CAG_64_ flies showed reduced retinal depth compared to WT flies, reflecting degeneration. When *stau* levels were then reduced by co-expressing *stau* shRNA, the retinal depth in flies was significantly improved (Fig. 4A–C). Transgenic upregulation of fly *stau* worsened the eye phenotype in hTDP-43 flies. Modulation of *stau* in WT flies had no effect on eye phenotype or retinal depth (Fig. 4D). These data indicate that Stau modulates neurotoxicity of hTDP-43 and hATXN2-CAG64 in *Drosophila*, supporting the potential for targeting STAU1 for treating neurodegenerative diseases.

### In vivo genetic interaction of overabundant STAU1 and TDP-43

**Fig. 5:**
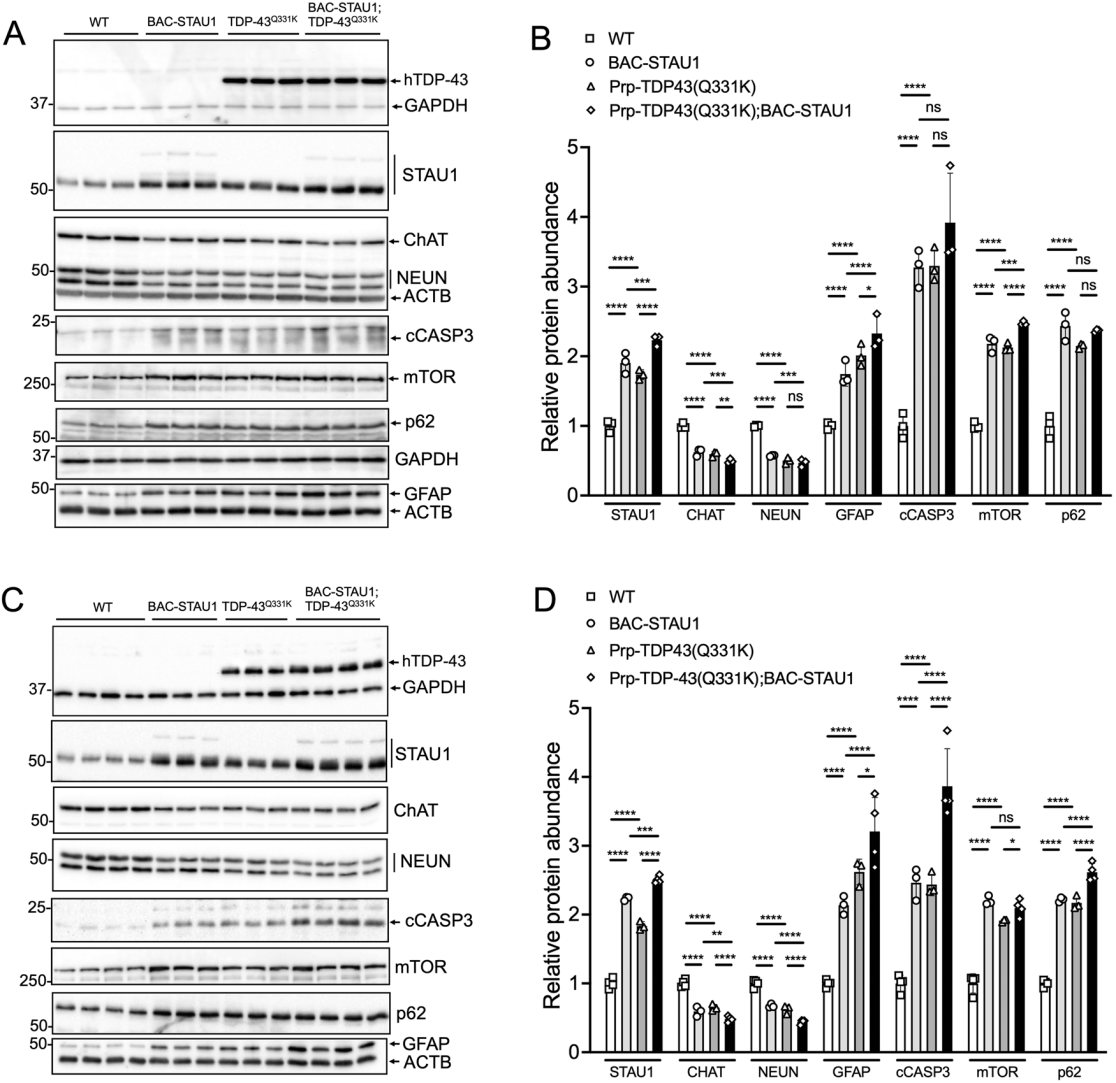
STAU1 genetic interaction with TDP-43. BAC-STAU1 mice were crossed with Prp-TDP43(Q331K) mice and brain hemisphere tissues from mice of each resultant genotype were evaluated by western blotting. Western blotting and quantification of the indicated proteins from brain of 8 wk old mice (**A**, **B**) and 24 wk old mice (**C**, **D**). Each lane represents an individual mouse, *N* = 3–4 mice per group. Means ± SD of biological replicates are plotted; the SD of technical replicates ranged from 0.13–1.2 (**B**), 0.15 to 1.2 (**D**). ns, non-significant; *, *p* < 0.05; **, *p* < 0.01; ***, *p* < 0.001; ****, *p* < 0.0001, two-way ANOVA with Bonferroni post hoc correction.

Previously we demonstrated that STAU1 is elevated in cerebellum and spinal cord of Thy1-TDP-43 transgenic mice and in sporadic ALS patient spinal cords, associated with abnormal abundance or phosphorylation of molecular markers of autophagy [6, 7]. Because autophagy and other neuronal markers are abnormally expressed in BAC-STAU1 mice (Fig. 3), we now sought to demonstrate that adding the expression of human STAU1 to TDP-43 transgenic mice would further worsen the molecular phenotype. To do so we performed a genetic cross of BAC-STAU1 mice with Prp-TDP43(Q331K) mice described in Wils et al. [46]. We then evaluated brain hemisphere tissues from mice of all resultant genotypes for the expression of ChAT, NeuN, cleaved CASP3, mTOR, p62 and GFAP, at 8 and 24 wks of age. Significant changes were observed for each of these proteins in BAC-STAU1 mice and Prp-TDP43(Q331K) mice in both age groups (Fig. 5). Additionally, nearly all of the proteins were significantly more elevated (STAU1, GFAP, cCASP3, mTOR, p62) or reduced (ChAT, NeuN) in double-transgenic Prp-TDP43(Q331K);BAC-STAU1 vs single-transgenic BAC-STAU1 mice. This was true in both age groups, with a few exceptions (cCASP3 and p62 in the 8 wk group, and mTOR in the 24 wk group) (Fig. 5). In general, the in vivo overexpression of hSTAU1 by crossing BAC-STAU1 mice with Prp-TDP43(Q331K) mice resulted in a significantly more severe molecular phenotype in either age group investigated. Note that in Fig. 5 the TDP-43 antibody used recognized only human TDP-43, and full blots using an antibody recognizing both mouse and human TDP-43 is shown in Supplemental Fig. 6A, B. Mouse TDP-43 was significantly elevated in BAC-STAU1 brain vs WT by 20–30% (Supplementary Fig. 6C). For all other full blots in the study see Supplementary Fig. 7.

### Artificial microRNAs targeting STAU1 improve the abnormal molecular phenotype in BAC-STAU1 mice

**Fig. 6:**
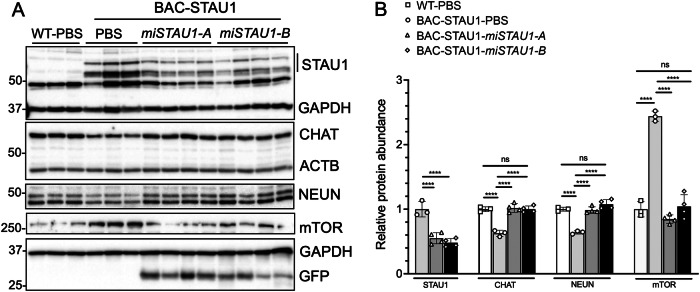
Knockdown of *STAU1* in BAC-STAU1 mice with an artificial microRNA normalizes ChAT, NeuN and mTOR abundance in spinal cord. BAC-STAU1 mice 20 wks of age were treated with 9.6E10 VG of AAV-*miSTAU1-A* or AAV-*miSTAU1-B* by single ICV injections. Control mice were treated with PBS vehicle. Mice were sacrificed at 23 wks of age and spinal cord proteins were evaluated by western blotting (**A**). Quantifications of three replicate blots demonstrated 50% reduction of STAU1 in SC, and that mice treated with either of the miRNAs had normalized levels of ChAT, NeuN and mTOR compared to levels in WT mice (**B**). The lower STAU1 band is non-specific to the antibody. Each lane represents a different mouse, *N* = 3–4 mice. Means ± SD of biological replicates are plotted; the SD of the technical replicates ranged from 0.03 to 0.37. ****, *p* < 0.0001, two-way ANOVA with Bonferroni post hoc correction.

As STAU1 overabundance in BAC-STAU1 is associated with abnormal autophagy and neuronal abundance markers, and lowering Stau in the fly improved the retinal depth neurodegenerative phenotype in TDP-43 and ATX2 flies, we sought to determine if lowering STAU1 abundance in BAC-STAU1 mice could improve these features. To do this we generated two different AAVs that deliver artificial microRNAs targeting human *STAU1*, designated AAV-miSTAU1-A and AAV-miSTAU1-B. We treated mice at 20 wks of age which was well after phenotype onset observed in Fig. 3A–F, and sacrificed mice at 23 wks of age. We then removed spinal cords and evaluated proteins by western blotting and quantification (Fig. 6A, B). Very little STAU1 was observed in WT mice while a high abundance of STAU1 was observed in BAC-STAU1 mice, as well as significantly reduced abundances of ChAT and NeuN, and normalized abundance of mTOR. BAC-STAU1 mice treated with AAVs were positive for GFP on blots, expressed by an independent promotor, indicating positive uptake of the AAVs. BAC-STAU1 mice that were treated with either of AAV-miSTAU1-A or AAV-miSTAU1-B had significantly reduced STAU1 abundance and normalized ChAT, NeuN, and mTOR compared to the BAC-STAU1 mice treated with vehicle. Levels of CHAT, NEUN and mTOR in the AAV-treated BAC-STAU1 mice were close to those observed in the WT mice (Fig. 6).

## Discussion

RNA-binding proteins (RBPs) including STAU1 play important roles in health and disease ranging from viral defense to neurodegeneration [47]. Pathogenic variants in some RBPs such as TDP-43 and FUS have been reported in patients with ALS and FTD, directly linking the respective genes to neurodegenerative diseases (NDDs). Overabundance of TDP-43, its subcellular mislocalization, and cytoplasmic aggregation are also typical in patients without pathogenic variants in ALS genes and TDP-43 aggregates are found in >90% of spinal cords of individuals with sporadic ALS. Although *STAU1* mutations have not yet been identified in NDDs, the STAU1 protein is overabundant in cells and spinal cord tissue from ALS patients and in ALS model systems as a result of a negative post-transcriptional feedback loop on autophagy [9].

Here we examined whether changes seen in vitro in the presence of STAU1 overabundance are replicated in vivo. We describe a new BAC-STAU1 mouse that expresses the entire human *STAU1* gene. The transgene was expressed in cerebral hemispheres, cerebellum, and spinal cord (Fig. 3) as well as in cultures of mouse neurons, astrocytes and microglia (Supplemental Fig. 1). Mouse *Stau1* transcript abundance was reduced in astrocyte cultures of BAC-STAU1 mice (Supplemental Fig. 1G). This pattern parallels the autoregulatory suppression of endogenous mouse TDP-43 observed in human TDP-43 transgenic mice [48]. Western blot analysis indicated that the BAC transgene expressed full-length STAU1 (Fig. 3).

### Transcriptomics autophagy neuronal apoptosis

Based on our findings in vitro [3, 6, 7, 11], we analyzed BAC-STAU1 mice for molecular markers of autophagy function and neurodegeneration as well as for transcriptomic changes. Bulk RNA-seq provided surprisingly few DEGs in spinal cord of BAC-STAU1 mice at 25 and 57 wks-of-age (Fig. 2). In both age groups, the nonsense mediated decay regulator *Smg5* appeared as the most significantly elevated transcript, supporting a role for STAU1 in NMD. Pathway analyses revealed a common signature of transcriptomic dysregulation in both the 25 and 57 wk age groups, with more pathways annotated in the 57 wk group compared to the 25 wk group. Notable pathways included Unfolded Protein Response that we had previously shown dependent on STAU1 [11], and reduced Fatty Acid Metabolism that we previously observed reduced in SC of BAC-ATXN2-Q72 mice [49] which have overabundant STAU1 [3]. Other pathways that were significantly altered in one or both groups were also associated with STAU1 previously, including mTORC1 Signaling (linked to autophagy) [7], Epithelial Mesenchymal Transition [50], Oxidative Phosphorylation [51], and Adipogenesis, Myogenesis and Myc Targets [13, 52]. Disease relevance for STAU1 is further supported by significant annotations for pathways of Alzheimer’s Disease, Huntington’s Disease, and Parkinson’s Disease (Fig. 2C).

Semiquantitative WB analyses showed significant abnormalities in pathways involving key signaling cascades with abnormal expression of mTOR, p-mTOR, p62 and LC3-II consistent with reduced autophagic flux (Fig. 3). These findings were confirmed in extracts from cerebral hemispheres, cerebellum, and spinal cord pointing to the potential role of STAU1 overabundance in NDDs affecting distinct parts of the CNS.

We previously established a biomarker profile of PC-specific proteins (CALB1, RGS8, PCP2, PCP4, FAM107B) that show early and continuous decline in two SCA2 mouse models as proxies for PC health and function [3, 37, 53]. These proteins were also dysregulated in the cerebellum of BAC-STAU1 mice consistent with PC dysfunction (Fig. 3C, D).

We applied a similar approach to protein extracts of cerebral hemispheres and spinal cords (Fig. 3A, B, E, F). Protein analyses were consistent with autophagy dysfunction. Neuronal injury was evidenced by decreased expression of NeuN and ChAT. Cleaved caspase-3 was significantly increased consistent with apoptotic cell death. As typically seen in NDDs, there was a significant increase in GFAP expression. Of note, ChAT abundance was not only decreased in spinal cord as a sign of motor neuron degeneration, but also in cerebral hemispheres consistent with degeneration in basal forebrain cholinergic neurons and potential relevance for memory dysfunction in AD and limbic-predominant age-related TDP-43 encephalopathy (LATE).

We also examined abundance of UNC13A and STMN2 in spinal cords. Previous studies have shown that TDP-43 mislocalization to the cytoplasm (and loss of nuclear splicing regulation) is associated with the inclusion of cryptic exons in human cells resulting in reduced expression of UNC13A and STMN2 [45, 54, 55]. Even though the cryptic exons described in humans have not been observed in mice, the abundances of both proteins were reduced in BAC-STAU1 mice, suggesting that their regulation, at least in mice, involves a mechanism other than the canonical human cryptic exon pathway—such as transcriptional or translational changes, potentially via STAU1-mediated decay. An alternative possibility is that the altered abundance of UNC13A and STMN2 reflects previously undescribed, TDP-43–dependent splicing abnormalities in mice. While the specific human cryptic exons are not conserved, murine TDP-43 pathology is known to induce aberrant splicing, including cryptic exon–like events [56, 57], raising the possibility of mechanistically analogous pathways.

The molecular changes seen in BAC-STAU1 mice were specific and reversible by RNAi. We developed 2 artificial miRNAs to human *STAU1* and delivered them by ICV injection. Both constructs worked equally well and reverted marker proteins back to levels seen in WT mice (Fig. 6). These findings are in line with our previous observations that genetic reduction of *Stau1* improves mouse SCA2 phenotypes [3] and support the development of therapeutics targeting STAU1 for treating neurodegenerative disease.

### Modulation of Staufen dosage

As reducing *Stau1* dosage protected cerebellar Purkinje cells and improved motor behavior in an SCA2 mouse model, the question arises whether increasing STAU1 abundance would worsen NDD phenotypes. We therefore examined the effects of staufen dosage in the fly and in a TDP-43 mouse model. Flies expressing human TDP-43 or polyglutamine expanded human ATXN2 (ATXN2-CAG_64_) were treated with shRNAs targeting the endogenous fly *stau* gene. The decreased retinal depth seen in flies transgenic for hTDP-43 or hATXN2-CAG_64_ was significantly improved by co-expressing *stau* shRNA (Fig. 4). Conversely, upregulation of fly *stau* further worsened the degenerative phenotype seen in the TDP-43 line, but not in the ATXN2 line.

We next investigated the role of Staufen in a mammalian system by crossing BAC-STAU1 mice with Prp-TDP-43(Q331K) transgenic mice, approximately doubling the amount of endogenous Staufen protein. Interestingly, while Mitchell et al. (2015) [48] reported reduced endogenous TDP-43 in this same transgenic line, we observed a 10–20% increase in endogenous murine TDP-43 at both 8 and 24 weeks (Supplemental Fig. 6C). We speculate that this difference may reflect a pathogenic autoregulatory response sensitive to chronic TDP-43 disfunction, as well as minor methodological differences between the studies. In Prp-TDP-43(Q331K) mice, neuronal marker proteins, glial and apoptotic markers are abnormal already at 8 wks of age, prior to the reported onset of motor abnormalities (Fig. 5, round symbols). Increasing STAU1 abundance worsened the levels of all marker proteins (ChAT, NeuN, cleaved CASP3, mTOR, p62 and GFAP) in brain with changes increasing from 8 to 24 weeks-of age (Fig. 5, diamond symbols). Of note, cleaved caspase-3 was 3.5-fold increased in double-transgenic mice compared to a 2.5-fold increase in single transgenic mice. These studies suggest that variability in STAU1 levels in model systems and possibly also in humans can modify neurodegeneration. Genetic and environmental factors affecting STAU1 abundance deserve further study.

### Implications for human disease

Despite the pronounced molecular phenotype in BAC-STAU1 mice including activation of apoptosis, we did not see a progressive motor phenotype, at least for the observation period of up to 1 year and the motor tests used in our studies (grip strength, rotarod) (Supplemental Figs. 3 and 4). It is quite likely that longer observation periods are needed and that more nuanced behavioral tests need to be applied. Similar dissociations between molecular pathology and overt motor or survival phenotypes have been widely reported in mouse models of TDP-43, tau, α-synuclein/Parkinson’s disease, and C9ORF72 [46, 48, 58–61].

The sensitivity of NDD phenotypes to levels of STAU1 protein may have implications for mouse models of human NDDs as well as for the human disease. Based on our studies it is reasonable to predict that genetic or environmental variation affecting STAU1 levels will affect NDD phenotypes. Mouse background is known to affect NDD phenotypes [62], but little is known about CNS STAU1 levels in different mouse strains. Large-scale sequencing projects comparing individuals with ALS to controls so far have not identified *STAU1* Mendelian pathogenic variants or suggested risk variants [63] although analyses searching for protective variants have received less attention [64]. It may be that STAU1 variation is not a modifier of disease risk itself, but age-of-onset or rate of progression. It is also possible that the role of STAU1 in early development may limit the extent of genetic variation and that genetic or epigenetic variation of genes acting on STAU1 play a role. Finally, it is possible that environmental factors such as brain trauma affect STAU1 levels, as has been shown for stress granule formation in the fly [65].

## Conclusions

Our findings in the BAC-STAU1 mouse model demonstrate that STAU1 overabundance alone at levels found in neurodegenerative mouse models is sufficient to impair autophagy and to promote apoptotic cell death in the brain. Moreover, altering STAU1 levels in the presence of disease-associated mutations influences the severity of the resulting phenotypes. These results strengthen the case for STAU1 as a therapeutic target in human neurodegenerative disorders.

## Supplementary information

Dataset 1

All supplemental figures and legends

Supplemental Table 1

Supplemental Table 2

Supplemental Table 3

Supplemental Fig 7 Original Data Full Blots

## Data Availability

The data that support the findings of this study are available on request from the corresponding authors (SMP and DRS).
