# Supplementary material for: A human Staufen1 BAC transgenic mouse exhibits abnormal autophagy and neurodegeneration across the central nervous system
Source: Cell Death Dis. 2026 May 14;17(1):620. doi: 10.1038/s41419-026-08830-x (PMC13342298; doi:10.1038/s41419-026-08830-x)
Supplement: Supplementary file 2 — All supplemental figures and legends [file 41419_2026_8830_MOESM2_ESM.pdf]

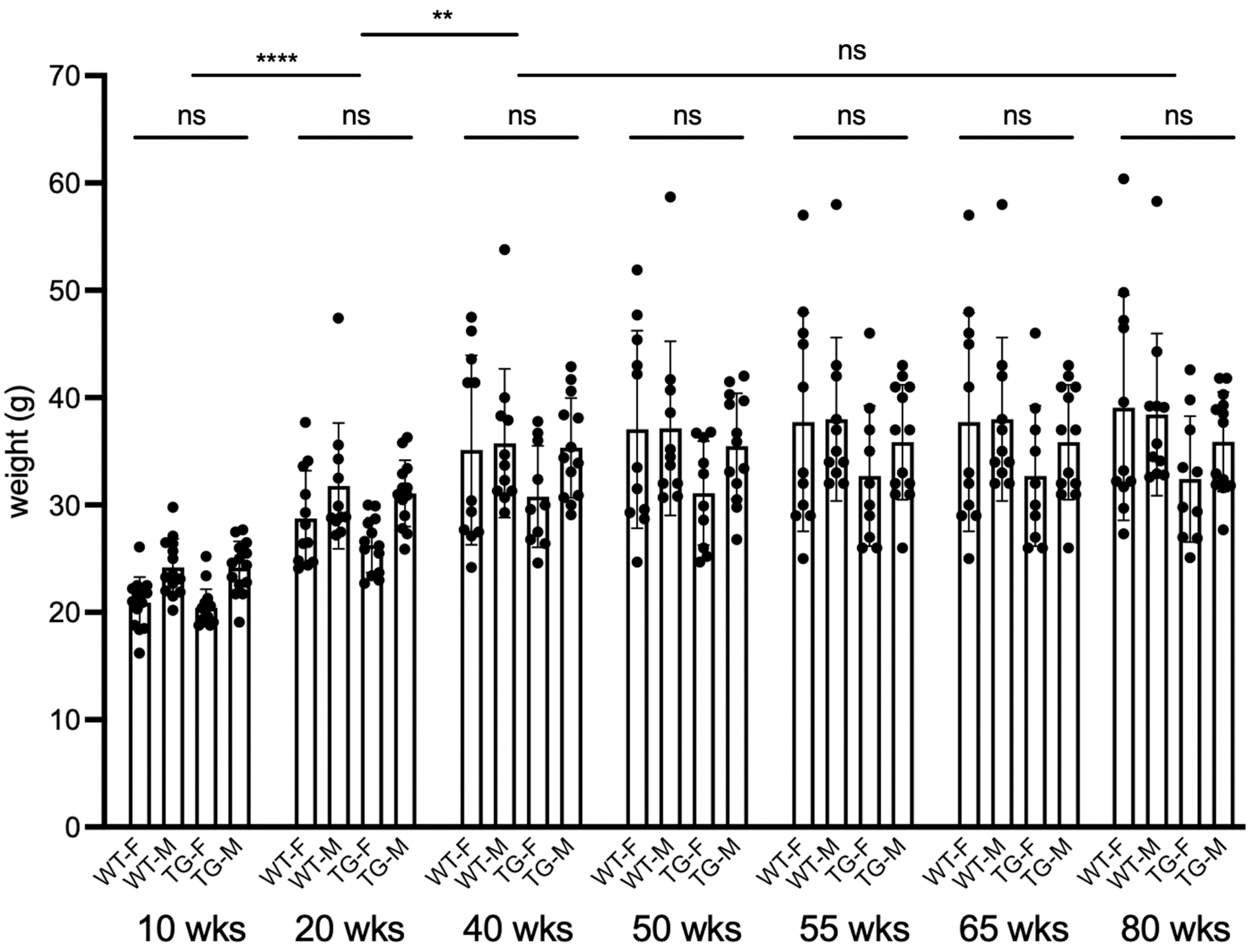

Supplemental Fig. 1. BAC-STAU1 mouse weights by sex and age. Shown are weights of female and male WT and BAC-STAU1 (TG) mice for 7 age groups over 80 wks. N = 11-14 mice (WT-F and WT-M), 10-16 mice (TG-F), 13-14 mice (TG-M). Two outlier mice remain in the chart that were 5.7 (WT-F) and 7.7 (WT-M) greater than the SD in the 80th wk. ns, non-significant; **, $p<0.01$; ****, $p<0.0001$, two-way ANOVA with Bonferroni post-hoc correction.

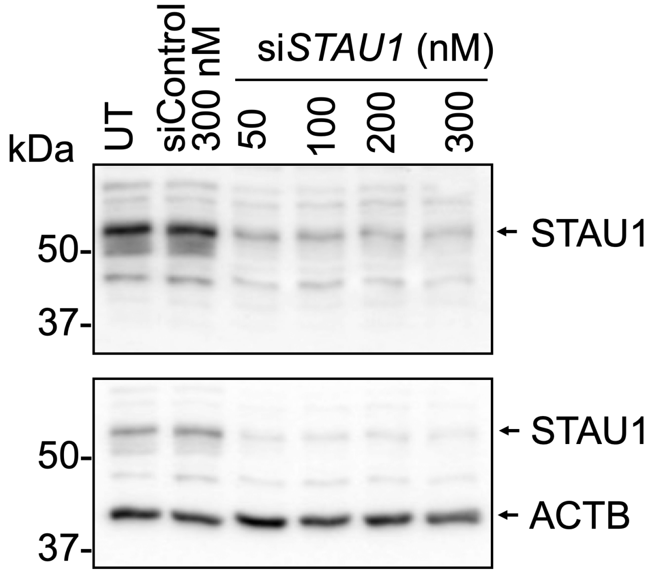

Supplemental Fig. 2. Characterization of anti-STAU1 antibody. HEK-293 cells were transfected with a control siRNA (siControl) and an siRNA targeting human STAU1 (siSTAU1), at the indicated doses, and harvested at 4 days posttransfection. Protein extracts were analyzed by western blotting to verify Staufen1 silencing. β-Actin was used as a loading control. According to the NCBI database, STAU1 is translated as multiple variants. Staufen antibody detects human STAU1.

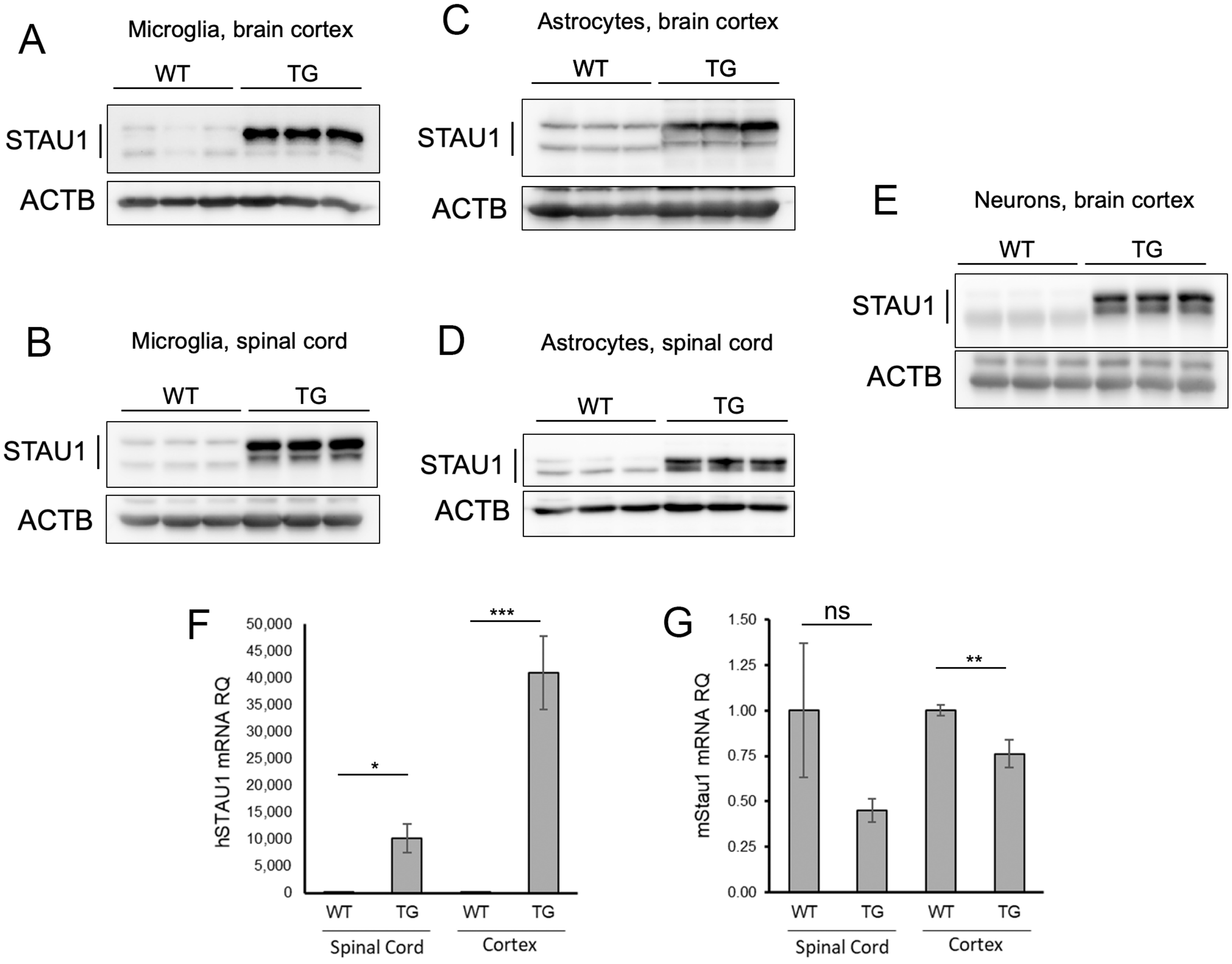

Supplemental Fig. 3. Expression of STAU1 in cultured cells of the brain cortex and spinal cord of BAC-STAU1 mice. Spinal cord cultures were prepared from neonate BAC-STAU1 mice (TG) or wildtype littermates (WT), cultured for 3 wks with conditions enriching for microglia and astrocytes, or for production of neurons. A-E) Western blotting: STAU1 was highly abundant in microglia of the cortex and SC (A&B), in astrocytes of cortex and SC (C&D), and cortical neurons (E). In A-E each lane represents one culture from different mice. F-G) qPCR: Human *STAU1* (F) and mouse *Stau1* (G) detected in cultured SC astrocytes and cultured cortical astrocytes. RQ, relative quantity to *Actb*. ns, non-significant; *, $p<0.05$; **, $p<0.01$; ***, $p<0.001$, Student's *t*-test.

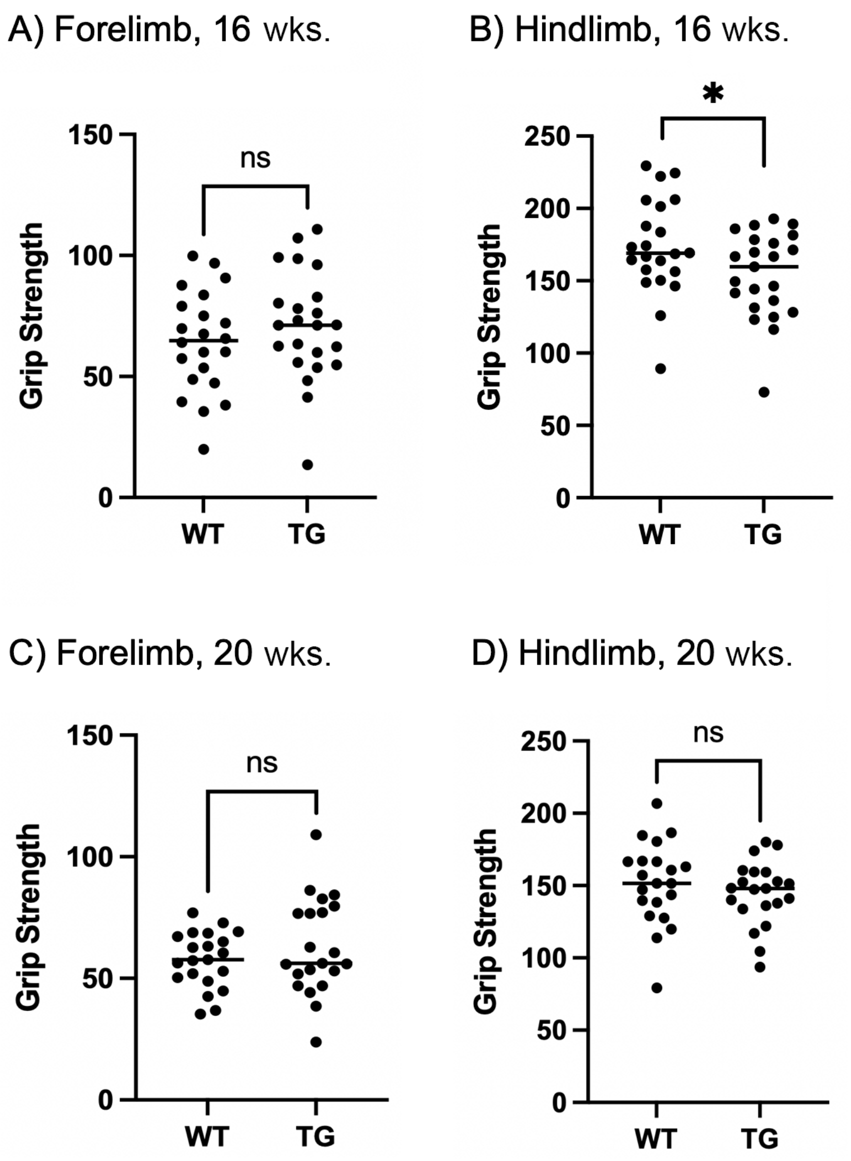

Supplemental Fig. 4. Grip strength testing. BAC-STAU1 mice were tested for forelimb and hindlimb strength at ages 16 wks (A,B) and 20 wks (C,D). Significant difference was observed for hindlimb strength at 16 wks (B), however statistical difference was not observed at 20 wks (D). N=22 WT & 23 TG (A,B) and 21 WT & 21 TG (C,D). Significance was determined using repeated measures ANOVA. Values shown are means of 3 replicates per mouse, and bars represent group means. ns, not significant; *, $p<0.05$.

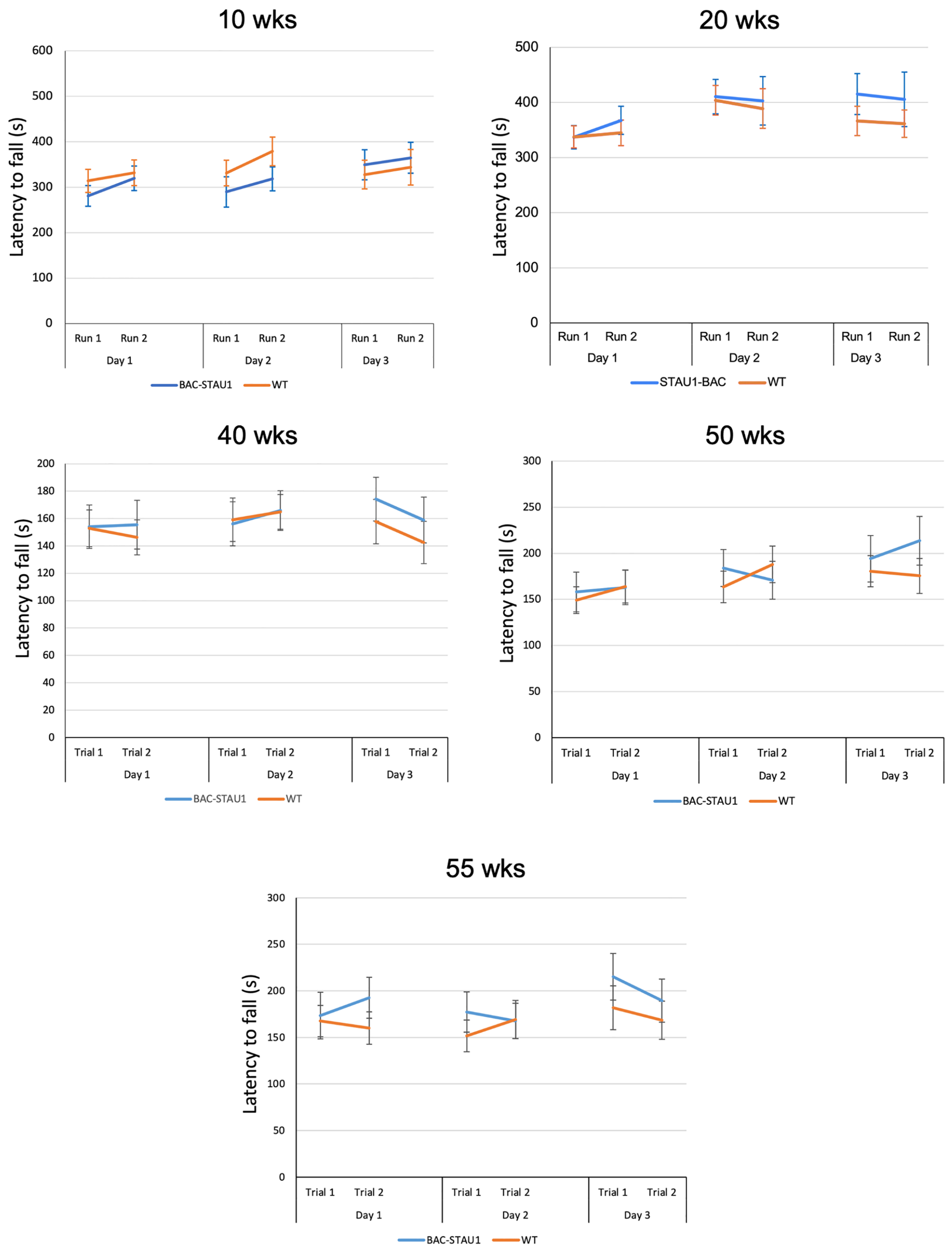

Supplemental Fig. 5. Rotarod behavioral testing. BAC-STAU1 mice and wildtype littermates were tested on the rotarod in 5 timepoints as indicated. Testing was performed in duplicate over three days in a week of testing. Values shown are mean ±SEM. No significant differences were observed (repeated measures ANOVA). N= 28 WT & 30 TG (10 wks), 23 WT & 27 TG (20 wks), 22 WT & 23 TG (40, 50, 55 wks).

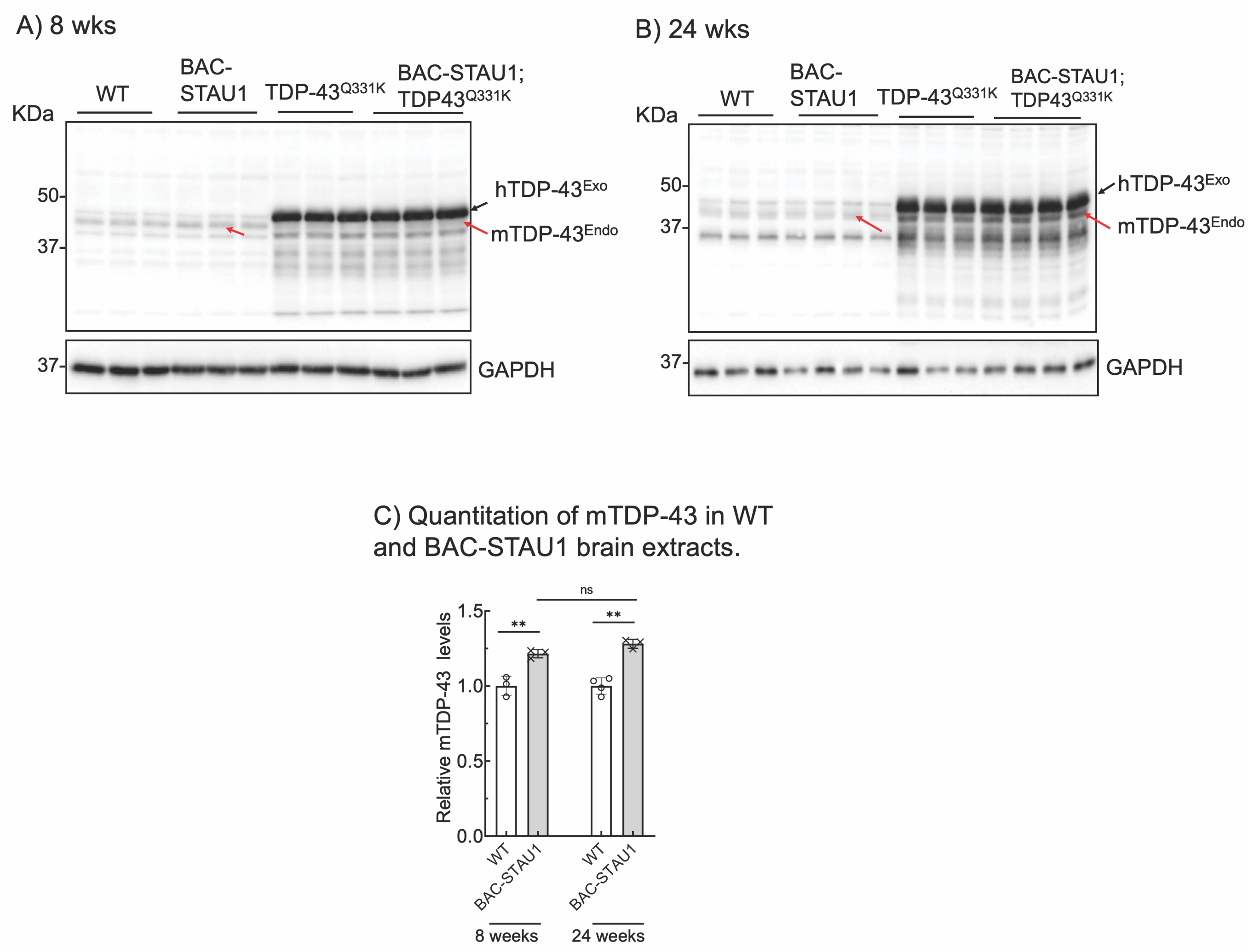

Supplemental Fig. 6. STAU1 genetic interaction with TDP-43. Western blots corresponding to those in Fig. 5 but using an antibody that recognizes both mouse TDP-43 (mTDP-43) and human TDP-43 (hTDP-43). As in Fig. 5, BAC-STAU1 mice were crossed with Prp-TDP43(Q331K) mice and brain hemisphere tissues from mice of each resultant genotype were evaluated by western blotting. Western blotting and quantification of the indicated proteins from brain of 8 wk old mice (A) and 24 wk old mice (B), and quantifications of mTDP-43 (C). Each lane represents an individual mouse, N=3-4 mice per group. Means ± SD of biological replicates are plotted; the SD of technical replicates ranged from 0.01-1.0 (8 wks), 0.01 to 0.06 (24 wks).. ns, non-significant; **, $p<0.01$, two-way ANOVA with Bonferroni post-hoc correction.
