# Supplementary material for: A human Staufen1 BAC transgenic mouse exhibits abnormal autophagy and neurodegeneration across the central nervous system
Source: Cell Death Dis. 2026 May 14;17(1):620. doi: 10.1038/s41419-026-08830-x (PMC13342298; doi:10.1038/s41419-026-08830-x)

Supplemental Fig. 7. Full blots. Presentation of full-blots for western blot experiments.

Fig. 3A (Brain, 14 wks of age)

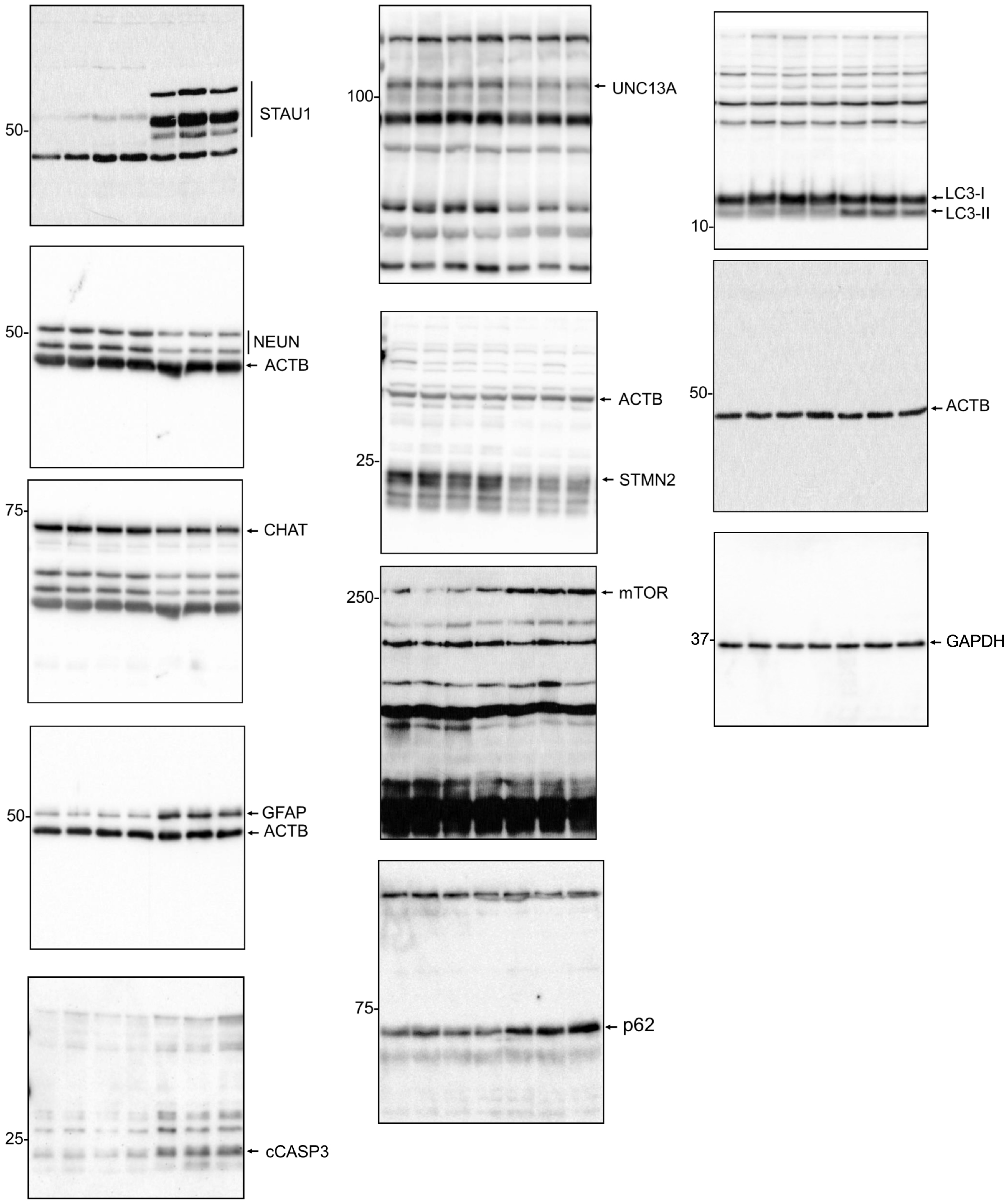

Fig. 3C (Cerebellum, 14 wks of age)

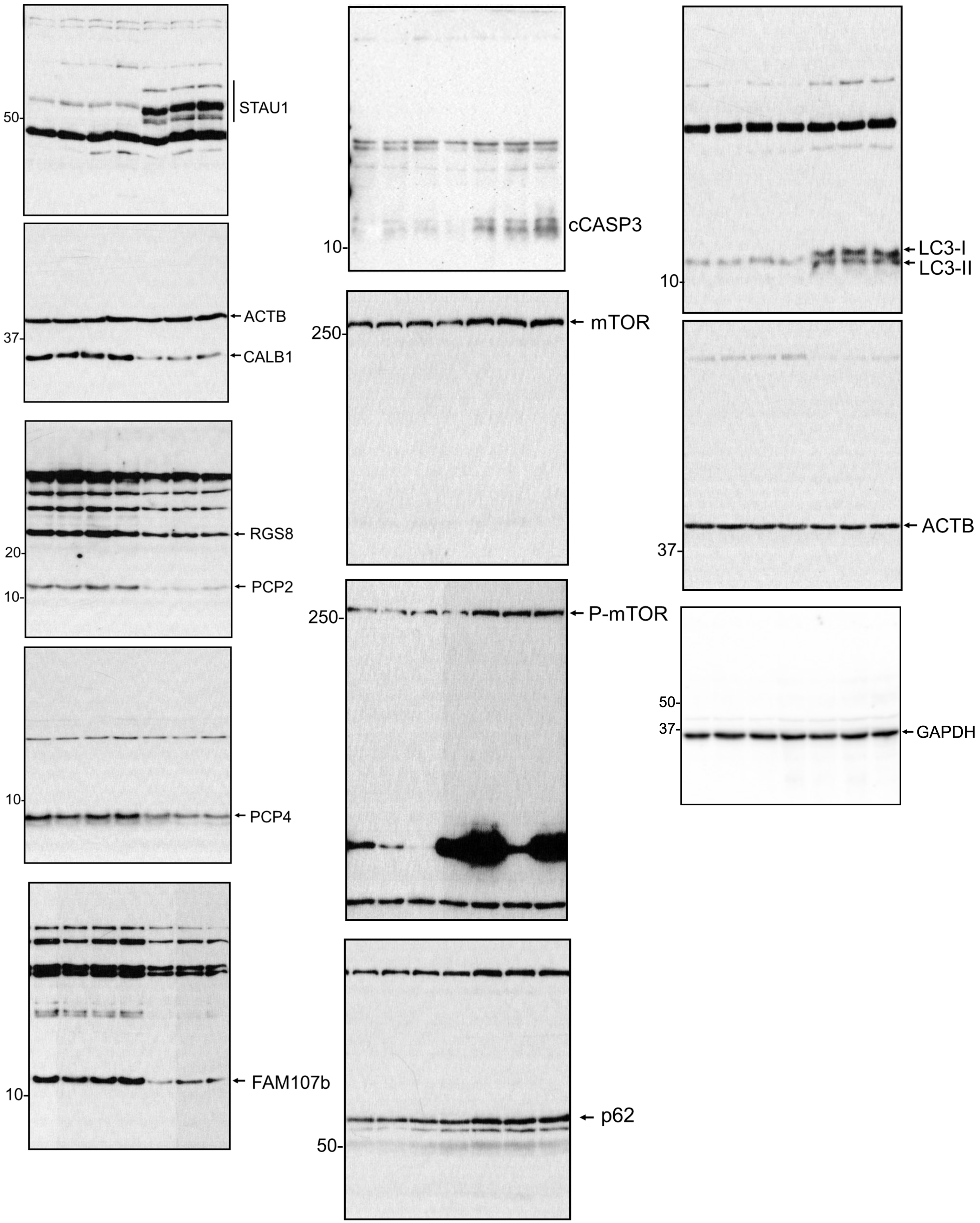

Fig. 3E (Spinal cord, 14 wks of age)

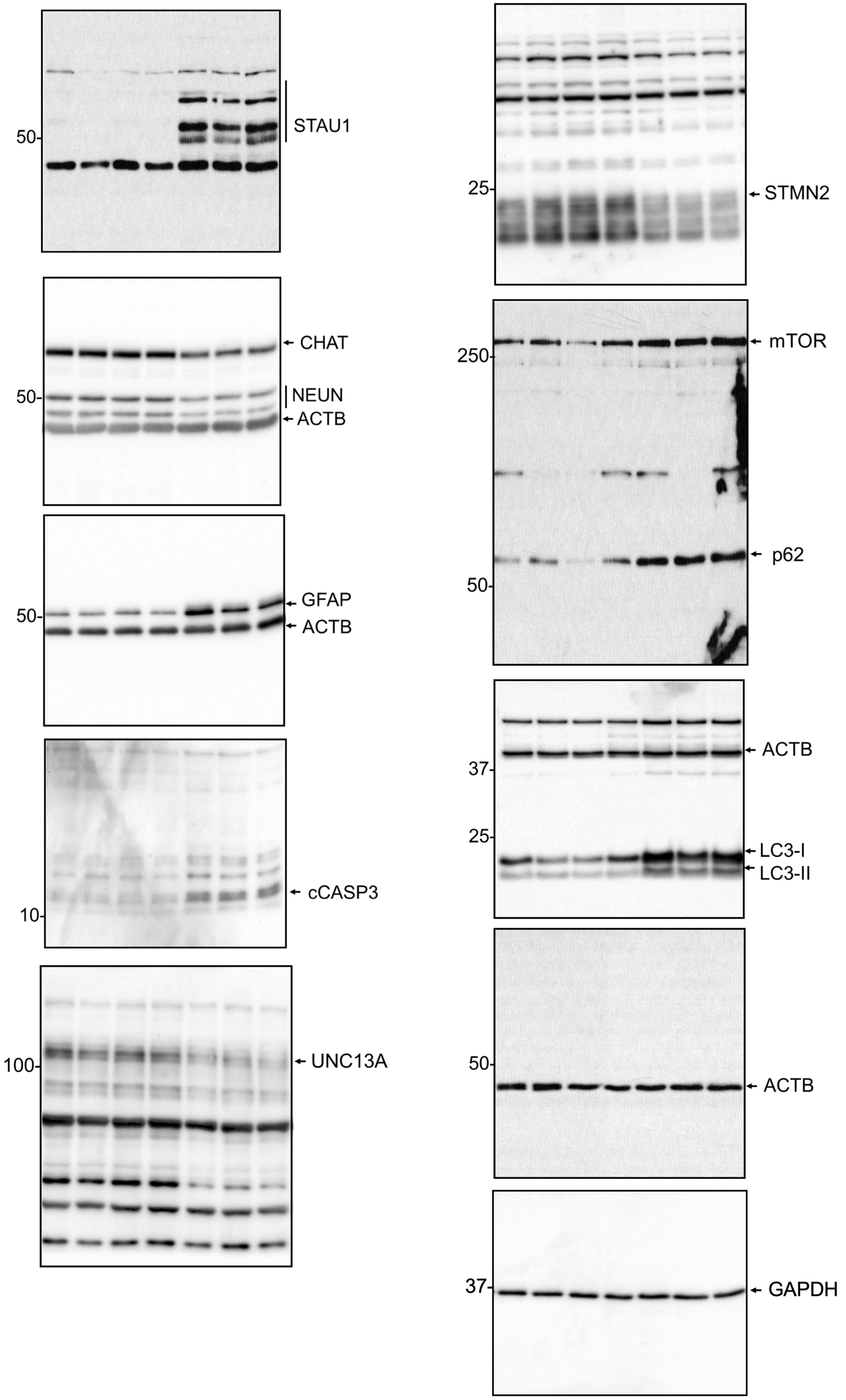

Fig. 5A, 8 wks

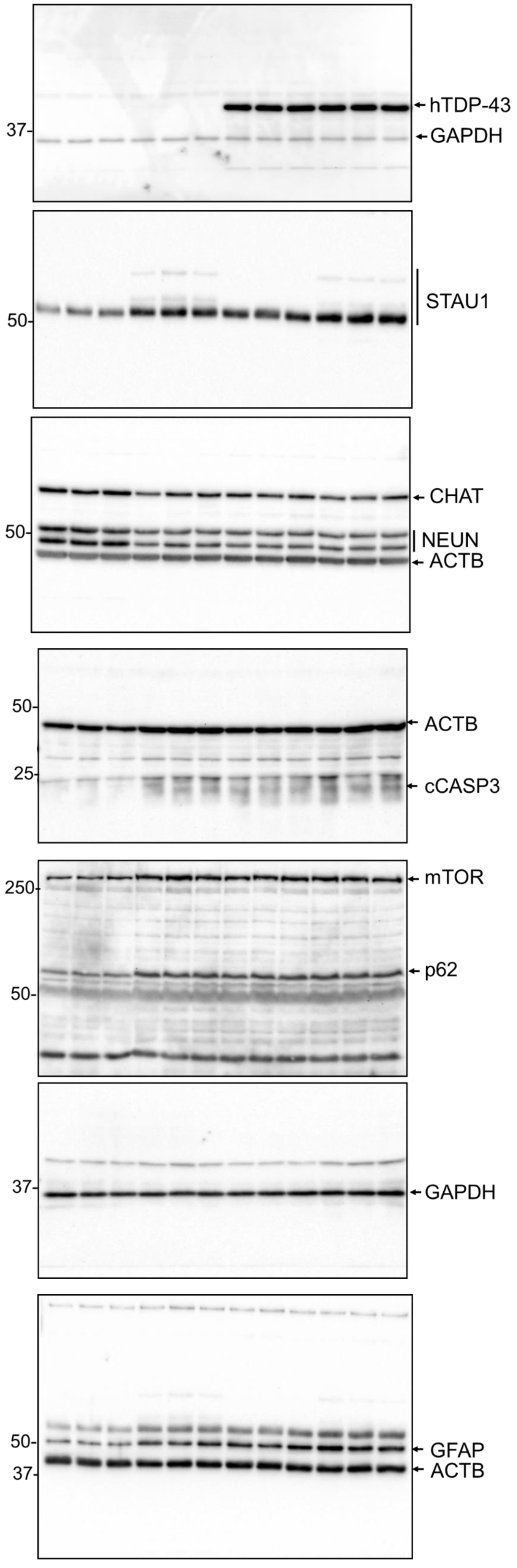

Fig. 5C, 24 wks

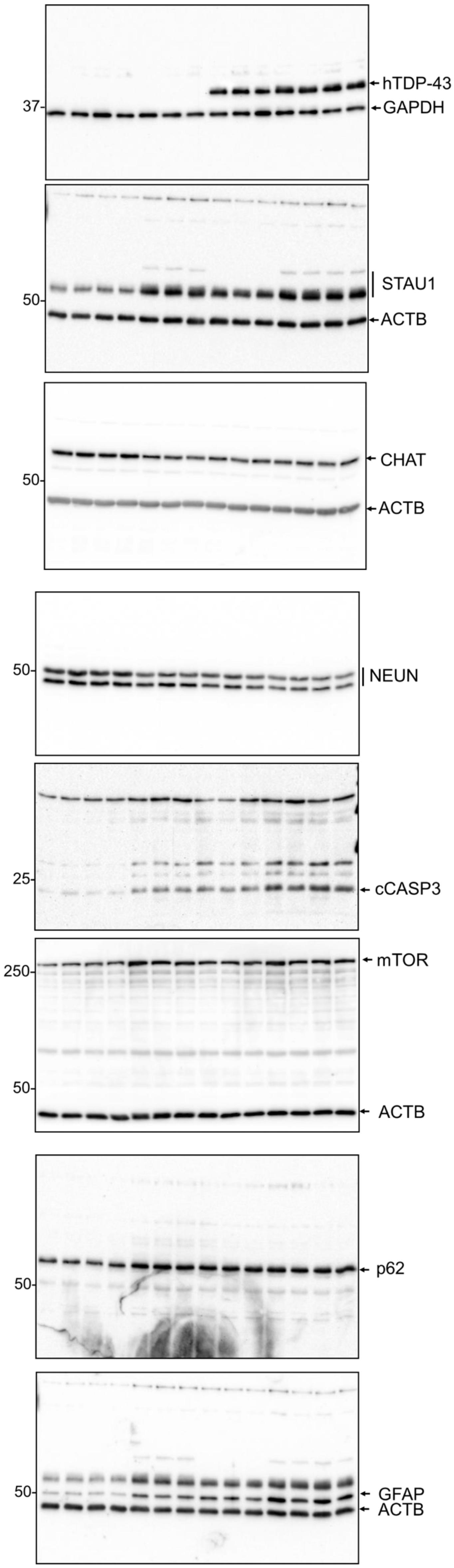

Fig. 6A

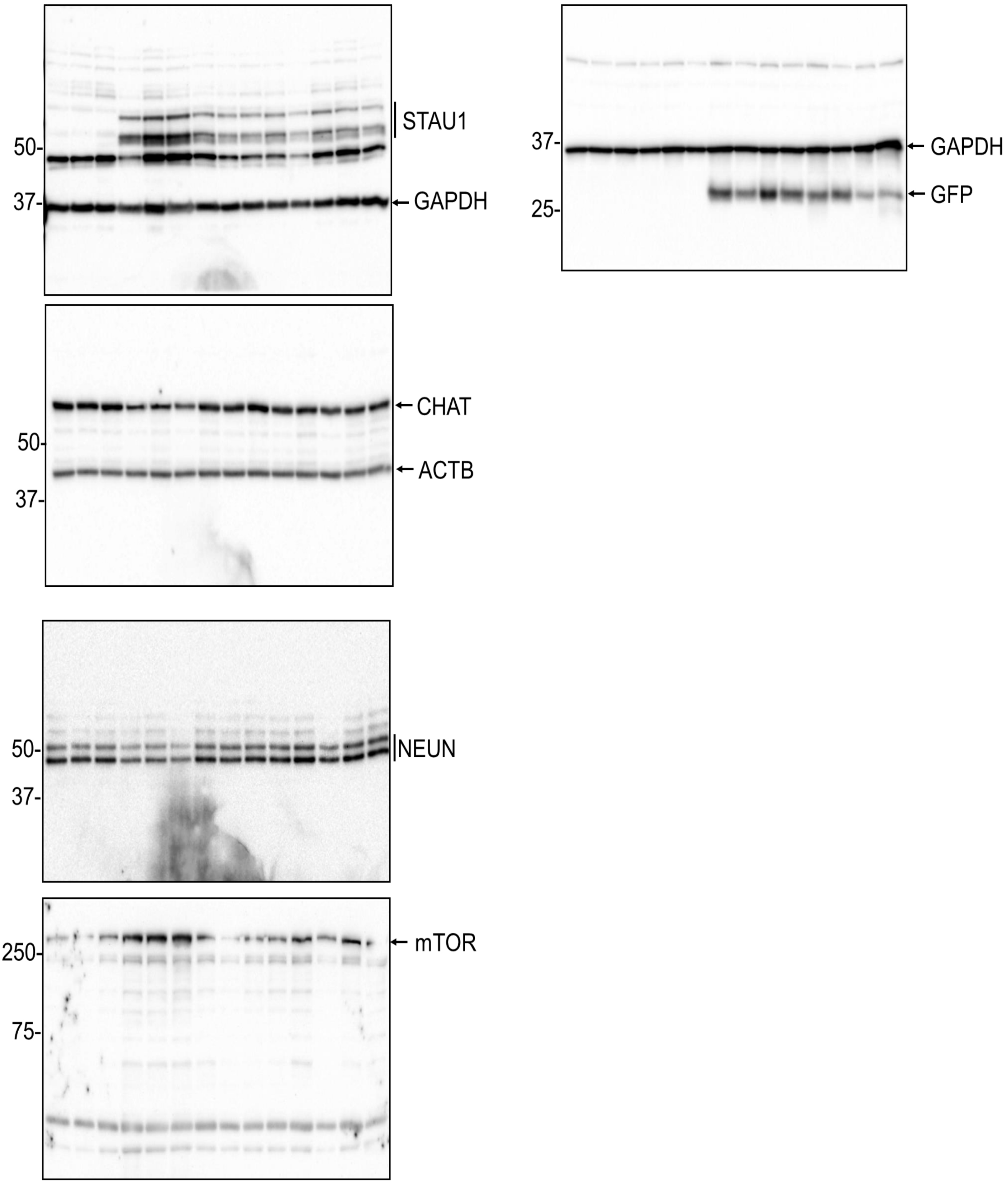

STAU1
50-
GAPDH
37-
CHAT
50-
ACTB
37-
NEUN
50-
37-
mTOR
250-
75-
37-
GAPDH
GFP
25-

Supplementary Fig. 2.

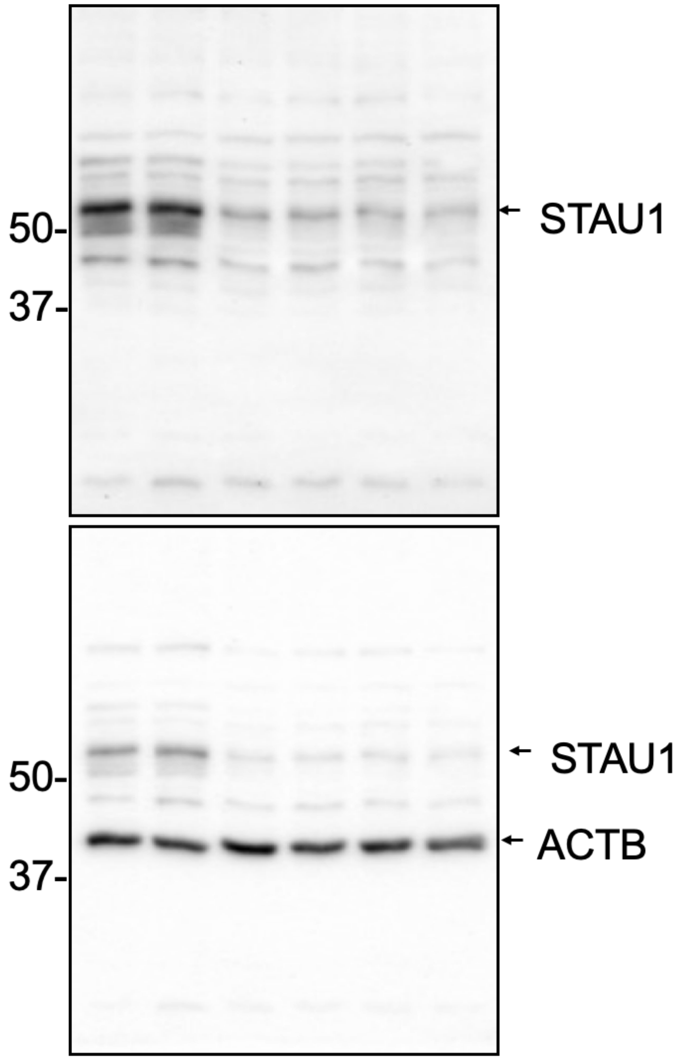

Supplemental Fig. 3

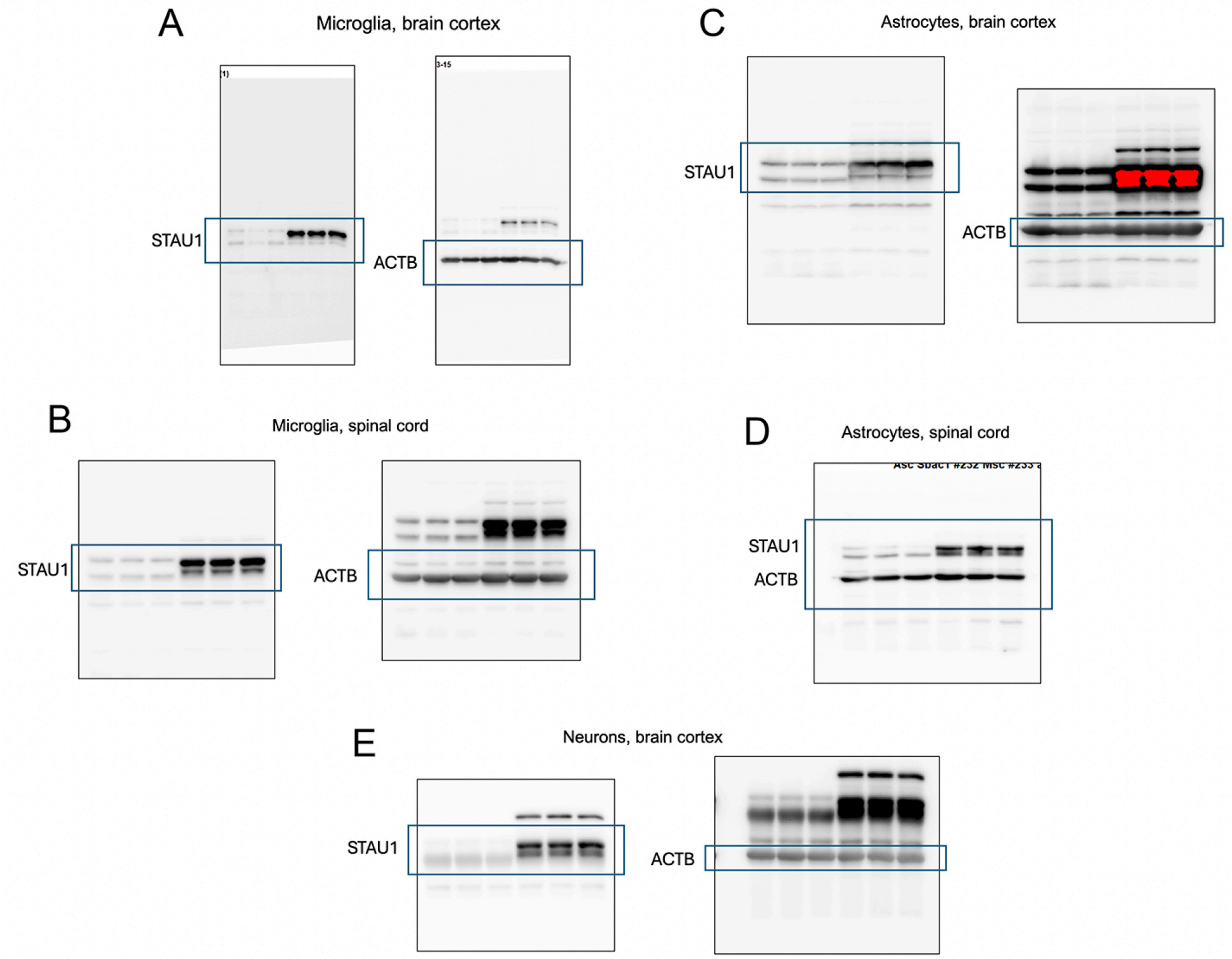

Supplemental Fig. 6.

A) 8 wks

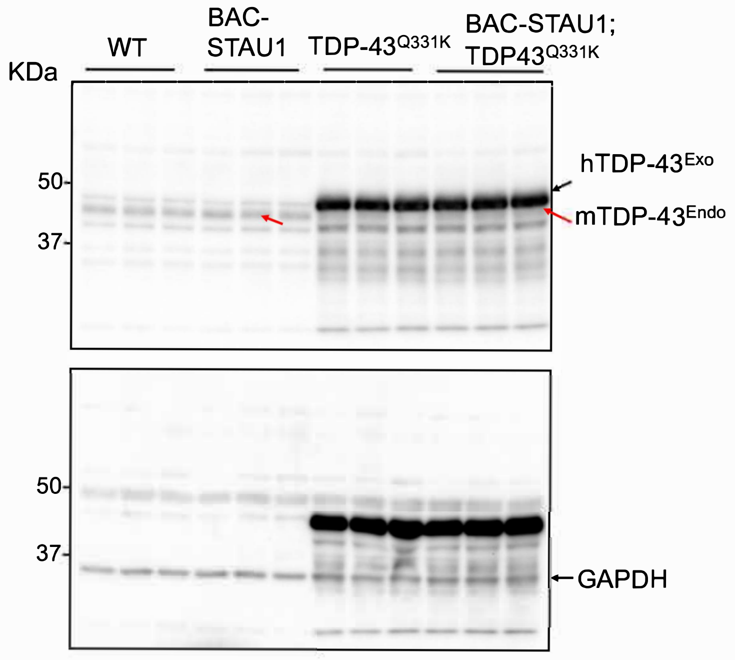

B) 24 wks

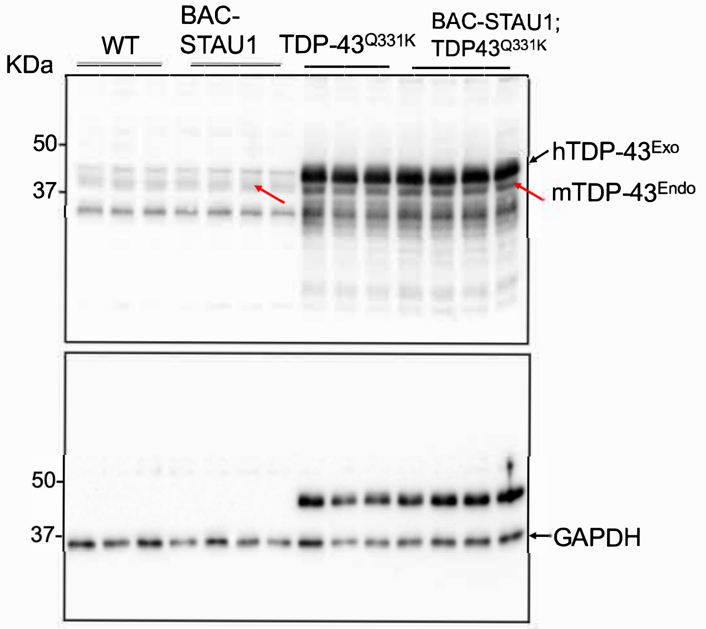

Supplement: Supplementary file 6 — Supplemental Fig 7 Original Data Full Blots [file 41419_2026_8830_MOESM6_ESM.pdf]
